# Pyrimidine Pool Disequilibrium Induced by a Cytidine Deaminase Deficiency Inhibits PARP-1 Activity, Leading to the Under Replication of DNA

**DOI:** 10.1371/journal.pgen.1005384

**Published:** 2015-07-16

**Authors:** Simon Gemble, Akshay Ahuja, Géraldine Buhagiar-Labarchède, Rosine Onclercq-Delic, Julien Dairou, Denis S. F. Biard, Sarah Lambert, Massimo Lopes, Mounira Amor-Guéret

**Affiliations:** 1 Institut Curie, Centre de Recherche, Orsay, France; 2 CNRS UMR 3348, Stress Génotoxiques et Cancer, Centre Universitaire, Orsay, France; 3 Institute of Molecular Cancer Research, University of Zurich, Zurich, Switzerland; 4 Université Paris Diderot, Sorbonne Paris Cité, Unité de Biologie Fonctionnelle et Adaptative (BFA) UMR 8251 CNRS, Plateforme Bioprofiler Bâtiment Buffon, 346A Case 7073, Paris, France; 5 CEA, DSV, iMETI, SEPIA, Fontenay-aux-Roses Cedex, France; University of Washington School of Medicine, UNITED STATES

## Abstract

Genome stability is jeopardized by imbalances of the dNTP pool; such imbalances affect the rate of fork progression. For example, cytidine deaminase (CDA) deficiency leads to an excess of dCTP, slowing the replication fork. We describe here a novel mechanism by which pyrimidine pool disequilibrium compromises the completion of replication and chromosome segregation: the intracellular accumulation of dCTP inhibits PARP-1 activity. CDA deficiency results in incomplete DNA replication when cells enter mitosis, leading to the formation of ultrafine anaphase bridges between sister-chromatids at “difficult-to-replicate” sites such as centromeres and fragile sites. Using molecular combing, electron microscopy and a sensitive assay involving cell imaging to quantify steady-state PAR levels, we found that DNA replication was unsuccessful due to the partial inhibition of basal PARP-1 activity, rather than slower fork speed. The stimulation of PARP-1 activity in CDA-deficient cells restores replication and, thus, chromosome segregation. Moreover, increasing intracellular dCTP levels generates under-replication-induced sister-chromatid bridges as efficiently as PARP-1 knockdown. These results have direct implications for Bloom syndrome (BS), a rare genetic disease combining susceptibility to cancer and genomic instability. BS results from mutation of the *BLM* gene, encoding BLM, a RecQ 3’-5’ DNA helicase, a deficiency of which leads to CDA downregulation. BS cells thus have a CDA defect, resulting in a high frequency of ultrafine anaphase bridges due entirely to dCTP-dependent PARP-1 inhibition and independent of BLM status. Our study describes previously unknown pathological consequences of the distortion of dNTP pools and reveals an unexpected role for PARP-1 in preventing DNA under-replication and chromosome segregation defects.

## Author Summary

The maintenance of genome stability is essential for the accurate transmission of genetic information, to ensure the successful duplication of chromosomes and their even segregation during mitosis. Errors occurring during DNA replication may affect both the accuracy of chromosome duplication and the balance of chromosome segregation during mitosis. Accurate DNA replication is strongly dependent on deoxynucleotides (dNTP) concentrations. Distortions in dNTP pool affect the rate of replication fork progression and compromise genetic stability. In the work presented here, we identified a novel mechanism by which dNTP pool disequilibrium compromises the completion of DNA replication and thus chromosome segregation, independently of the rate of fork progression. This mechanism involves the intracellular accumulation of deoxycytidine due to cytidine deaminase (CDA) deficiency, inhibiting PARP-1 activity. These results have direct implications for Bloom syndrome (BS), a rare genetic disease combining susceptibility to cancer and genomic instability. BS cells also have a CDA defect, resulting in a high frequency of ultrafine anaphase bridges due entirely to dCTP-dependent PARP-1 inhibition. These data highlight new pathological consequences of the distortion of dNTP pools and reveal an unexpected role for PARP-1 in preventing the accumulation of excessive amounts of unreplicated DNA and chromosome segregation defects.

## Introduction

DNA replication is a fundamental cellular process that ensures duplication of the genetic information and subsequent transfer to daughter cells. The accuracy of DNA replication can be hampered by various exogenous and endogenous stresses, threatening genome integrity. It has become clear that replication stress, due to disturbance of the DNA replication program, is a major source of genome instability early during cancer development. Replication stress is defined as any phenomenon that alters the fulfillment of the DNA replication program. These phenomena include alteration of the initiation and elongation steps of DNA replication, conflicts between DNA replication and metabolic pathways such as transcription and mRNA processing, nucleotide pool disequilibrium, and overexpression or activation of oncogenes [1–4]. Some loci in the human genome are particularly difficult to replicate. They include common fragile sites (CFSs) that have a high A-T content and origin-poor regions, and such loci are prone to the formation of secondary structures and late replication [5, 4]. Such "difficult to replicate" regions are very sensitive to replication stress. Such stress can jeopardize the completion of their replication, with the possibility of the formation of intertwined sister chromatid bridges during mitosis [6].

There are two types of sister-chromatid anaphase bridges: chromatin bridges, consequences of defective sister chromatid segregation [7], can be stained with conventional dyes, such as DAPI; and ultrafine anaphase bridges (UFBs), which cannot be stained with conventional DNA dyes or antibodies against histones [8, 9]. UFBs were discovered only recently, and are generally identified by the detection of the helicase-like protein, PICH (Plk1-interaction checkpoint “helicase”). They have been found in all cultured normal cells tested, and are therefore probably physiological structures [10]. However, their prevalence often increases in constitutive or induced replication stress conditions, such as Bloom syndrome and the inhibition of replication progression, respectively. [9, 11, 12] As cells progress through anaphase, UFBs are progressively stretched and decrease in number, as they are resolved. Most UFBs detected in normal cells are of centromeric origin: they are thought to contain unresolved DNA catenations between the centromeres separating during anaphase [9]. However, some UFBs, of common fragile site origin [11, 13] (CFS-associated UFBs), are induced by treatment with the replication inhibitor aphidicolin and are detected through the binding of FANCD2/FANCI protein complexes to bridge ends. Defects in either FANCD2 or its partner FANCI are involved in Fanconi anemia syndrome, and these proteins have been reported to co-localize with fragile sites [14]). UFBs, the origin and function of which remain unclear, do not result from recombination intermediates [15, 16]. They may correspond to replication intermediates persisting on entry into mitosis, reflecting a failure to complete DNA replication or to resolve sister chromatid catenanes fully [6, 17]. As CFS are replicated late and associated with loci poor in replication initiation events [18, 19], it has been suggested that CFS-associated UFBs originate from DNA incompletely replicated when the cell enters mitosis [11].

In cells from Bloom syndrome (BS) patients, chromatin bridges and PICH-positive UFBs are abnormally frequent [9]. The correlation between chromosomal instability and an increased risk of malignancy at an early age is stronger in BS than other pathological or physiological situations [20]. BS is a consequence of mutations in both copies of the *BLM* gene which encodes a 3’-5’ DNA helicase identified as a member of the RecQ family [21]. A characteristic of BLM-deficient cells is the frequency of sister chromatid exchange (SCEs) [22]. The BS cellular phenotype also includes chromosome breaks, slow replication fork speed, and high frequencies of both blocked replication forks and anaphase bridges [23, 24]. These cellular features reflect the presence of endogenous DNA damage, replication stress and chromosome segregation defects and implicate BLM in favoring faithful duplication of the genome. However, we and others have reported that BLM deficiency leads to the downregulation of cytidine deaminase (CDA) [25, 26], an enzyme of the pyrimidine salvage pathway. CDA catalyzes the hydrolytic deamination of cytidine and deoxycytidine (dC) to uridine and deoxyuridine (dU), respectively [27]. CDA deficiency causes excess dCTP leading to nucleotide pool disequilibrium. Some of the genetic instability associated with BLM deficiency, including the slow replication fork progression and about 30% of the increased sister chromatid exchange (SCE) frequency, results from a pyrimidine pool imbalance due to the CDA defect [25]. Whether other cellular defects associated with BS phenotype are due to CDA deficiency remains to be established.

Here, we investigated the mechanism underlying the increase in UFB frequency in BS cells and its possible relationship to pyrimidine pool disequilibrium. We demonstrate that the formation of supernumerary UFBs, but not chromatin bridges, is fully due to defective CDA and not to the BLM defect *per se*. The increase in the dCTP pool resulting from CDA defect leads to a significant reduction of basal PARP-1 activity. PARP-1 is a multifunctional protein involved in diverse physiological processes and in the response to DNA damage [28]. Lower basal PARP-1 activity causes higher frequencies of unreplicated centromeres, foci of mitotic DNA synthesis and (UFB)-containing unreplicated DNA, without replication fork speed being affected.

Our investigations reveal a novel mechanism of UFB formation and new pathological consequences of the distortion of dNTP metabolism fluxes. These findings imply that the effects of nucleotide pool disequilibrium may be more far-reaching than previously thought, and include jeopardizing genome stability not only through the regulation of fork progression, but also by reducing PARP-1 activity. We also provide evidence that PARP-1 has a previously unsuspected role in preventing replication stress and chromosome segregation defects.

## Results

### CDA prevents supernumerary UFB formation in BS cells and in cells expressing BLM

**Fig 1:**
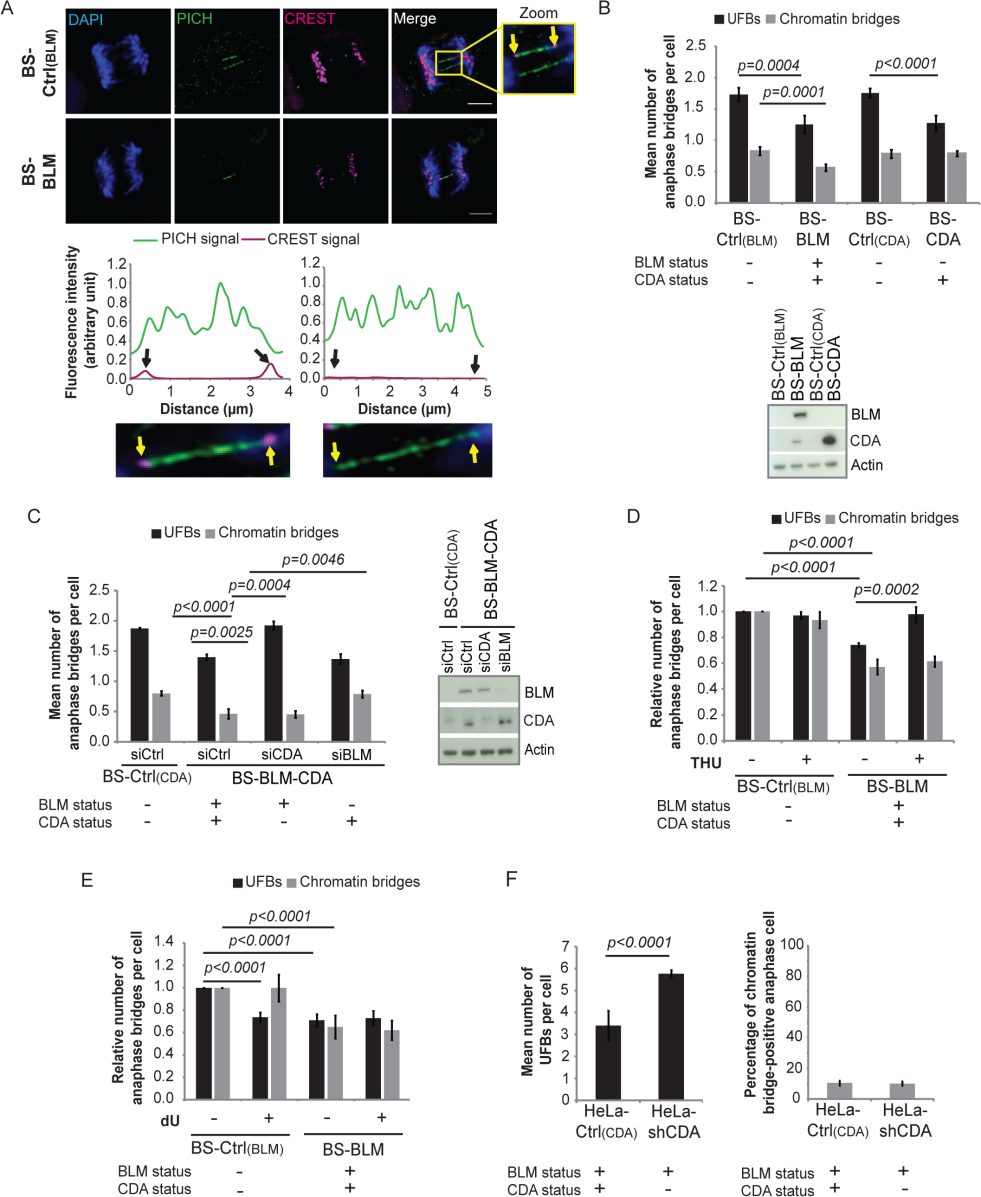
CDA prevents supernumerary UFB formation in BS cells and in cells expressing BLM. (A) Upper panel: Representative immunofluorescence deconvoluted z‐projection images of PICH-positive UFBs in BS-Ctrl_(BLM)_ and in BS-BLM anaphase cells. DNA was visualized by DAPI staining (blue). Centromeres were stained with CREST serum (in magenta) and UFBs were stained with PICH antibody (in green). In the enlarged image, CREST foci at the extremities of the UFB are indicated by yellow arrows. Scale bar: 5 μm. Lower panel: Fluorescence quantifications along UFBs using Image J. CREST-positive UFBs are characterized by CREST fluorescence signal at the extremities (left). CREST-negative UFBs present no CREST signal (right). (B) Mean number of UFBs (black bars) and chromatin bridges (gray bars) per anaphase cell in BS-Ctrl_(BLM)_, BS-BLM, BS-Ctrl_(CDA)_ and BS-CDA cell lines (upper panel); BLM and CDA levels, assessed by immunoblotting (lower panel). Errors bars represent means ± SD from five independent experiments (> 170 anaphase cells per condition (UFBs) or > 95 anaphase cells per condition (chromatin bridges)). (C) Mean number of UFBs (black bars) and of chromatin bridges (gray bars) per anaphase cell in BS-Ctrl_(CDA)_ cells and in BS-BLM-CDA cells transiently transfected with the indicated siRNAs (left panel); BLM and CDA levels, assessed by immunoblotting (right panel). Error bars represent means ± SD from three independent experiments (> 120 anaphase cells per condition (UFBs) or > 70 anaphase cells per condition (chromatin bridges)). (D-E) Relative numbers of UFBs (black bars) and of chromatin bridges (gray bars) per anaphase cell in BS-Ctrl_(BLM)_ and BS-BLM cell lines left untreated or treated with (D) 100 μM tetrahydrouridine (THU) or (E) 100 μM deoxyuridine (dU). Error bars represent means ± SD from: (D) five independent experiments (> 140 anaphase cells per condition (UFBs) or > 95 anaphase cells per condition (chromatin bridges)), (E) five independent experiments (> 120 anaphase cells per condition (UFBs) or > 95 anaphase cells per condition (chromatin bridges)). (F) Mean number of UFBs per cell (left panel), and percentage of chromatin bridge-positive anaphase cells (right panel) in HeLa-Ctrl_(CDA)_ and HeLa-shCDA cells. Error bars represent means ± SD from six independent experiments (> 155 anaphase cells per condition (UFBs) or > 100 anaphase cells per condition (chromatin bridges)). The significance of differences was assessed with Student’s *t*-test.

We investigated whether the higher frequency of anaphase bridges in BLM- and CDA-deficient cells derived from BS patients (BS cells, BLM-/CDA-) was due to CDA deficiency itself. The characteristics of all the cell lines used in this study are presented in Table 1. The expression of GFP-BLM in BS cells (BS-BLM) restores the expression of both BLM and CDA (BLM+/CDA+), as well as nucleotide pool equilibrium [25]. We analyzed the frequencies of chromatin bridges and UFBs in these cells by DAPI and PICH antibody staining, respectively (Fig 1A). The numbers of both chromatin bridges and UFBs were lower in BS-BLM cells (BLM+/CDA+) than in control cells (BS-Ctrl_(BLM)_, BLM-/CDA-), as previously reported [9]. The stable overexpression of CDA in BS cells (BS-CDA, BLM-/CDA+) restored the nucleotide pool equilibrium but did not restore BLM expression [25]; the frequency of UFBs in these cells was similar to that in BS-BLM cells (BLM+/CDA+), whereas the frequency of chromatin bridges remained similar to that observed in BS control cells (BS-Ctrl_(CDA)_, BLM-/CDA-) (Fig 1A and 1B). UFBs originating from the centromere and CFS were similarly affected in CDA-deficient cells (BS-Ctrl_(BLM)_ and BS-Ctrl_(CDA)_) (Fig 1A and S1A Fig). CDA expression in BS cells (BS-BLM and BS-CDA) significantly increased the percentage of cells with no UFBs and substantially decreased the frequency of cells with more than three UFBs, indicating that the entire BS cell population is affected by CDA deficiency (S1B and S1C Fig). These results suggest that supernumerary UFBs in BS cells result from the CDA defect and that the increase in chromatin bridge frequency may result directly from BLM deficiency.

**Table 1:** Characteristics of cell lines.

Cell lines	Origin of cell lines	Antibiotics	Integrated vectors	BLM status	CDA status	PARP-1 status	References
**BS-Ctrl**_**(BLM)**_	GM8505B (derived from BS patient)	G418	EGFP-C1	-	-	+	[25]
**BS-BLM**			EGFP-C1-BLM	+	+	+	
**BS-Ctrl**_**(CDA)**_	BS-Ctrl(BLM)	G418 + puromycin	EGFP-C1 + pCI	-	-	+	
**BS-CDA**			EGFP-C1 + pCI-CDA	-	+	+	
**BS-BLM-Ctrl**	BS-BLM	G418 + puromycin	EGFP-C1-BLM + pCI	+	+	+	In this study
**BS-BLM-CDA**			EGFP-C1-BLM + pCI-CDA	+	+	+	
**HeLa-Ctrl**_**(CDA)**_	HeLa	Puromycin	pGIPZ	+	+	+	
**HeLa-shCDA**			pGIPZ-shCDA	+	-	+	
**HeLa-Ctrl**_**(PARP-1)**_	HeLa	Hygromycin B	pEBV	+	+	+	[46]
**HeLa-shPARP-1**			pEBV-shPARP-1	+	+	-	

To verify these findings, we constructed cells stably expressing both BLM and CDA under the control of the CMV promoter (BS-BLM-CDA); this construct allowed the subsequent downregulation of BLM without downregulating CDA. In this model, CDA overexpression had no effect on the frequencies of chromatin bridges and UFBs (S1D Fig); however, siRNA-mediated CDA downregulation (BS-BLM-CDA-siCDA, BLM+/CDA-) increased UFB frequency to the levels observed in BS control cells (BS-Ctrl_(BLM/CDA)_-siCtrl, BLM-/CDA-), without affecting chromatin bridge frequency (Fig 1C). Also, siRNA-mediated BLM downregulation (BS-BLM-CDA-siBLM, BLM-/CDA+) increased the chromatid bridge frequency to that in control BS cells (BS-Ctrl_(CDA)_-siCtrl, BLM-/CDA-), without affecting UFB frequency (Fig 1C). Thus, BLM prevents increases in chromatin bridge frequency, whereas CDA prevents increases in UFB frequency. We used tetrahydrouridine (THU), a potent CDA inhibitor [29] that does not modify CDA levels, to confirm these findings (S1E Fig). THU treatment induced a 33% increase in UFB frequency in CDA-expressing cells (BS-BLM, BLM+/CDA+), without affecting chromatin bridge frequency, but did not affect frequencies of UFBs and chromatin bridges in CDA-deficient cells (BS-Ctrl_(BLM),_ BLM-/CDA-) (Fig 1D). Thus, the effect of THU, increasing UFB prevalence, was dependent on CDA, confirming that THU is a specific inhibitor of CDA, as previously reported [29]. BS cells (BS-Ctrl_(BLM)_, BLM-/CDA-) were cultured in the presence of dU (dU is the final product of CDA, so its addition mimics CDA activity [25] without modifying CDA expression; S1E Fig): UFB frequency was 29% lower in BS cells cultured with than without dU, whereas chromatin bridge frequency did not differ (Fig 1E). Supplementation with dU had no effect on UFB or chromatin bridge frequencies in CDA-expressing cells (BS-BLM, BLM+/CDA+) (Fig 1E). These findings demonstrate that the abnormally high frequency of chromatin bridges in cells from BS patients is caused by the BLM deficiency whereas that of UFBs is due to the CDA deficiency.

We confirmed these findings in another cellular model based on an adenoviral short hairpin RNA (shRNA) specific for CDA to downregulate CDA stably in HeLa cells (HeLa-shCDA). The CDA downregulation was highly efficient, with no detectable CDA protein and significantly less CDA mRNA than in controls (S1F Fig). The CDA depletion in this model resulted in dCTP accumulation, replication fork slowing and an increase in SCE frequency, reproducing the part of the BS phenotype associated with CDA deficiency [25] (S1G, S1H and S1I Fig). UFB frequency was 41% higher in HeLa-shCDA cells than in control cells, whereas chromatin bridge frequency was unaffected (Fig 1F). These results were confirmed in four additional independent HeLa-shCDA clones, to check that they were not due to a clonal artifact. The UFB frequency in CDA-depleted HeLa cells was decreased by 25% by dU treatment, whereas that in control HeLa cells was increased by 34% by THU treatment (S1J Fig). These experiments further confirm the role of CDA in preventing UFB formation in BLM-expressing cells.

Thus, all supernumerary UFBs in BS cells result from the CDA deficiency, and CDA prevents supernumerary UFB whether or not BLM is expressed.

### UFB formation does not result from DNA replication fork uncoupling

Our data indicate that a CDA defect, and thus pyrimidine pool disequilibrium, results in an increase in the prevalence of centromere and CFS-associated UFBs. Arlt and Glover (2010) showed that very low doses of camptothecin (CPT) greatly decreased the frequency of CFS gaps and breaks upon replication stress, and the amount of single-stranded DNA at stalled replication forks. On the basis of these results, the authors suggested that polymerase-helicase uncoupling is a key initial event in CFS instability. They suggested that the slowing of the replication fork might cause such uncoupling, generating genetic instability [30].

**Fig 2:**
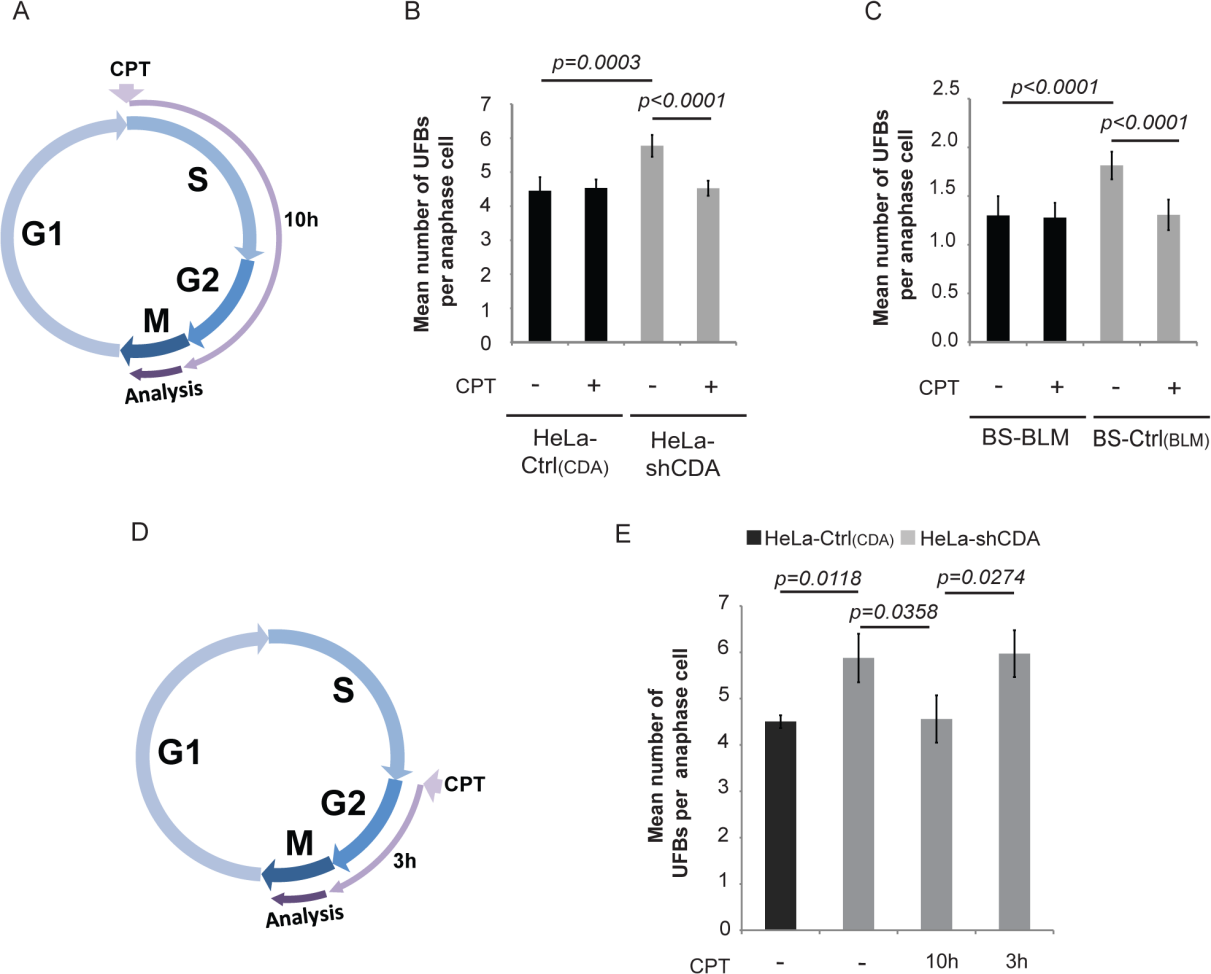
The formation of UFB-containing unreplicated DNA does not result from DNA replication fork uncoupling. (A) Schematic representation of 10 hours of treatment with 2 pM CPT during the cell cycle; only cells treated during the S and G2 phases were analyzed in anaphase. (B) Mean number of UFBs per anaphase cell, for HeLa-Ctrl_(CDA)_ (black bars) and HeLa-shCDA (gray bars) cells left untreated or treated with 2 pM CPT. Error bars represent means ± SD from six independent experiments (> 230 cells per condition). (C) Mean number of UFBs per anaphase cell, for BS-Ctrl_(BLM)_ and BS-BLM cells left untreated or treated with 2 pM CPT. Error bars represent means ± SD from six independent experiments (> 150 cells per condition). (D) Schematic representation of 3 hours of treatment with 2 pM CPT during the cell cycle; only cells treated during G2 phase were analyzed in anaphase. (E) Mean number of UFBs per anaphase cell in untreated HeLa-Ctrl_(CDA)_ (black bars) and in HeLa-shCDA (gray bars) cells left untreated or treated for 10 or 3 hours with 2 pM CPT. Error bars represent means ± SD from three independent experiments (> 90 anaphase cells per condition). The significance of differences was assessed with Student’s *t*-test.

We, therefore, first investigated whether very low doses of CPT could rescue UFB formation in the absence of CDA. We treated CDA-deficient cells and their corresponding CDA-expressing control cells with 2 pM CPT for 10 hours (S and G2 phases of the cell cycle, Fig 2A) or 3 hours (G2 phase; Fig 2D) [31]; we verified that this treatment did not affect CDA expression or the cell cycle (S2A and S2B Fig). In CDA-deficient cells, but not CDA-expressing cells, UFB frequency was significantly decreased by CPT treatment but only if it was administered during both the S and G2 phases (Fig 2B, 2C and 2E). Thus, as reported for CFS instability, treatment with 2 pM CPT during S-phase abolished the excess of UFBs in CDA-deficient cells.

We then investigated whether replication uncoupling could be responsible for UFB formation. Replication uncoupling is thought to result in long stretches of single-stranded DNA (ssDNA) [30] potentially detectable by electron microscopy (EM). We analyzed replication intermediates from CDA-depleted and control HeLa cells, treated or not treated with CPT, but observed no significant differences in the lengths of ssDNA at replication forks between the four conditions tested (S2C–S2E Fig). Thus, neither CDA depletion nor 2 pM CPT treatment induced detectable changes in the length of ssDNA stretches at replication forks. However, CDA-deficient cells displayed a marked accumulation of ssDNA gaps, visible on both replicated and parental duplexes (S2C and S2F Fig), consistent with impaired ssDNA gap repair in these cells. This is also consistent with our results showing a constitutive activation of both γH2AX and Chk2 in CDA-depleted HeLa cells (S2G Fig), as previously reported in BS cells [24]. Moreover, as for other conditions affecting the integrity of the replication template [32], the accumulation of ssDNA gaps in untreated CDA-depleted cells was associated with an increase in the frequency of reversed forks. As described for more acute CPT treatments [33, 34], 2 pM CPT treatment increased the frequency of both ssDNA gaps and reversed forks, regardless of CDA status (S2F and S2H Fig).

These findings suggest that the slowing of replication in CDA-depleted cells is not accompanied by generalized replication fork uncoupling, but rather with marked accumulation of ssDNA gaps and the associated increase in fork reversal. The accumulation of UFBs in CDA-deficient cells thus appears to be a consequence of other phenomena, which is suppressed by mild CPT treatment, through a molecular mechanism unrelated to fork uncoupling.

### CDA deficiency prevents the completion of DNA replication by the end of S phase, leading to the formation of UFB-containing unreplicated DNA

**Fig 3:**
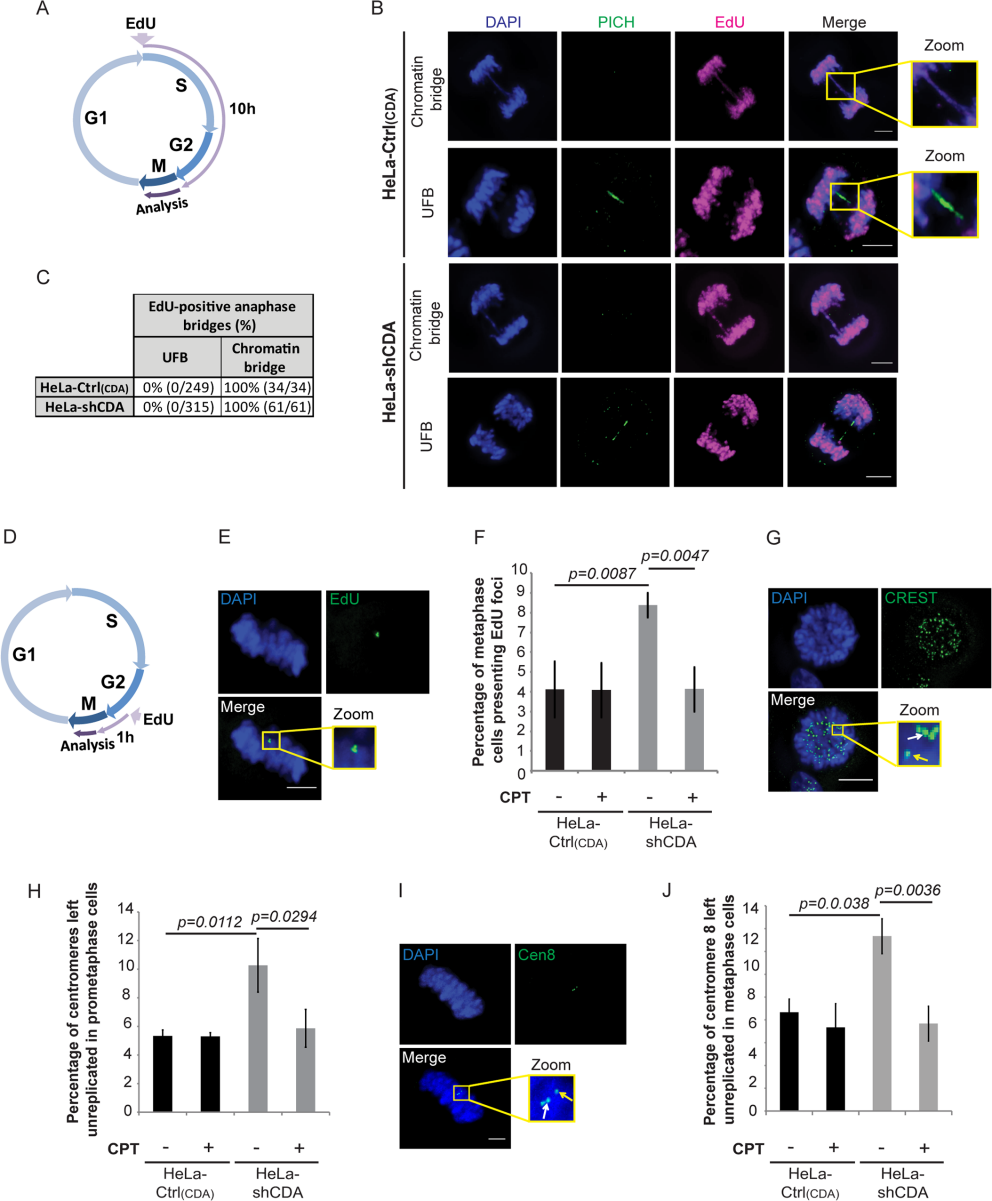
CDA deficiency impedes the completion of DNA replication by the end of S phase, leading to the formation of UFB-containing unreplicated DNA. (A) Schematic representation of 10 hours of EdU labeling during the cell cycle; only cells incorporating EdU during the S and G2 phases were analyzed in anaphase. (B) Representative immunofluorescence deconvoluted z‐projection images of HeLa-Ctrl_(CDA)_ and HeLa-shCDA cells showing EdU incorporation into chromatin bridges but not into PICH-positive UFBs. DNA was visualized by DAPI staining (blue). EdU was stained with Alexa Fluor 555 (in magenta) and UFBs were stained with PICH antibody (in green). Enlarged images show a chromatin bridge with EdU incorporation (upper panels) and a PICH-positive UFB with no EdU incorporation (lower panels). (C) Table showing the percentage of EdU incorporation into chromatin bridges and UFBs in HeLa-Ctrl_(CDA)_ and HeLa-shCDA cells. (D) Schematic representation of 1 hour of EdU labeling during the cell cycle; only cells incorporating EdU during late G2 and early mitosis were analyzed in metaphase. (E) Representative immunofluorescence deconvoluted z‐projection images of a metaphase HeLa-Ctrl_(CDA)_ cell with EdU incorporation. DNA was visualized by DAPI staining (blue). EdU was stained with Alexa Fluor 555 (in green). Enlarged images show three EdU foci on mitotic chromosomes. (F) Percentage of HeLa-Ctrl_(CDA)_ (black bars) and HeLa-shCDA (gray bars) metaphase cells left untreated or treated with 2 pM CPT and presenting EdU foci. Error bars represent means ± SD for three independent experiments (> 120 metaphase cells per condition). (G) Representative immunofluorescence deconvoluted z‐projection images of a prometaphase HeLa-Ctrl_(CDA)_ cell. DNA was visualized by DAPI staining (blue). Centromeres were stained with CREST serum (in green). Boxed images are enlarged; single-dotted CREST foci are indicated by yellow arrows and double-dotted CREST foci are indicated by white arrows. (H) Percentage of centromeres left unreplicated in HeLa-Ctrl_(CDA)_ (black bars) and in HeLa-shCDA (gray bars) prometaphase cells left untreated or treated with 2 pM CPT. Error bars represent means ± SD from three independent experiments (> 95 prometaphase cells per condition). (I) Representative immunofluorescence deconvoluted z‐projection images of a metaphase HeLa-Ctrl_(CDA)_ cell. DNA was visualized by DAPI staining (blue). Chromosome 8 centromeres were stained with the Cen-8 probe (in green). Boxed images are enlarged; single-dotted centromere 8 foci are indicated by yellow arrows and double-dotted centromere 8 foci are indicated by white arrows. (J) Percentage of chromosome 8 centromeres left unreplicated in HeLa-Ctrl_(CDA)_ (black bars) and in HeLa-shCDA (gray bars) metaphase cells left untreated or treated with 2 pM CPT. Error bars represent means ± SD from three independent experiments (> 115 metaphase cells per condition). Scale bar: 5 μm. Statistical significance was calculated with Student’s *t*-test.

We thus investigated the alternative molecular mechanism by which CDA deficiency contributes to supernumerary UFB formation. UFBs may be derived from unreplicated DNA or from double-stranded DNA catenanes [9]. UFBs cannot be stained by conventional DNA dyes, so we directly labeled DNA during the S and G2 phases of the cell cycle [31]. Cells were incubated for 10 hours with the thymidine analog 5-ethynyl-2’-deoxyuridine (EdU) and studied for bridges in anaphase (Fig 3A). In both CDA-depleted HeLa cells and control cells, EdU incorporation was detected in all chromatin bridges, but not in UFBs (Fig 3B and 3C). We cannot exclude the possibility that the thinness of UFB DNA structures limits EdU detection, but these data suggest that UFBs do not contain replicated DNA and might be derived from unreplicated DNA. We explored this hypothesis with other approaches.

The replication of some chromosomal loci, such as CFS, may be completed during late G2 or early mitosis [35, 36]. We thus investigated whether CDA deficiency could lead to detectable DNA synthesis during mitosis that would reflect under-replication of some chromosomal loci by the end of S phase. We therefore labeled CDA-depleted HeLa cells and control cells with EdU for 1 hour and analyzed the following mitosis (Fig 3D). EdU foci in mitosis were significantly more numerous in CDA-depleted than control HeLa cells (Fig 3E and 3F). About 55% of the EdU foci colocalized with CREST foci and about 24% of the EdU foci colocalized with FANCD2 foci, independently of the presence or absence of CDA (S3 Fig). Using the Image J macro “confined displacement algorithm” [37], we confirmed that the percentage of colocalization between EdU foci and CREST or FANCD2 foci was significantly different from that expected if the colocalization occurred by chance (*p*<0.05).

As the excess of UFBs was abolished by CPT treatment in CDA-depleted cells, we analyzed the effect of CPT treatment on mitotic DNA synthesis. We found that the CPT treatment of CDA-depleted HeLa cells also prevented the accumulation of mitotic cells presenting EdU foci (Fig 3F). Thus, both centromeres and CFSs are replicated particularly late in the absence of CDA, raising the possibility that some of these loci remain unreplicated when the cell enters mitosis. Importantly, CPT treatment abolished the excess of both mitotic DNA synthesis and UFBs in CDA-deficient cells, establishing a molecular link between the late replication of centromeres and CFSs, and UFB prevalence.

We then hypothesized that not all centromeres and CFS are fully replicated in cells lacking CDA when entering mitosis. We used CREST staining and FISH (fluorescence *in situ* hybridization)-based assay with a centromeric probe specific for chromosome 8 (Cen-8) to investigate whether centromere replication was impaired in CDA-deficient cells (double-dotted and single-dotted CREST or Cen-8 foci, indicating fully replicated and unreplicated centromeres, respectively). The frequency of unreplicated centromeres was significantly higher in prometaphase/metaphase CDA-depleted HeLa cells than in the corresponding control cells, further evidence that UFBs arise from unreplicated DNA (Fig 3G–3J). The CPT treatment of CDA-depleted HeLa cells also prevented the accumulation of unreplicated centromeres, indicating that defective centromere replication is likely a source of mitotic DNA synthesis and UFBs.

Altogether, these data suggest that in CDA-deficient cells, some “difficult-to-replicate sites”, such as centromeres and CFS, are replicated particularly late, or even left unreplicated, leading to UFB formation. DNA synthesis events during mitosis may help rescue the duplication of some of these loci. However, many may not be processed by mitotic DNA synthesis, resulting in UFB formation. Importantly, CPT treatment rescued the defect in centromere replication, mitotic DNA synthesis and the excess of UFBs, providing a molecular link between compromised replication at “difficult to replicate sites” and UFB formation.

### CDA deficiency is associated with weak basal PARP-1 activity, leading to incomplete replication of DNA, promoting UFB formation

We investigated how CPT treatment counteracts the effects of CDA deficiency. CPT and other genotoxic agents activate PARP-1 (Poly(ADP-ribose) polymerase-1), a multifunctional protein preventing genetic instability in response to DNA damage and replication stress [28, 38]. PARP-1 catalyzes poly(ADP-ribosyl)ation (PARylation) by transferring ADP ribose units from nicotinamide (NAD+) onto diverse acceptor proteins [39]: this post-translational modification is important in a wide array of physiological processes and in response to DNA damage [40–42].

**Fig 4:**
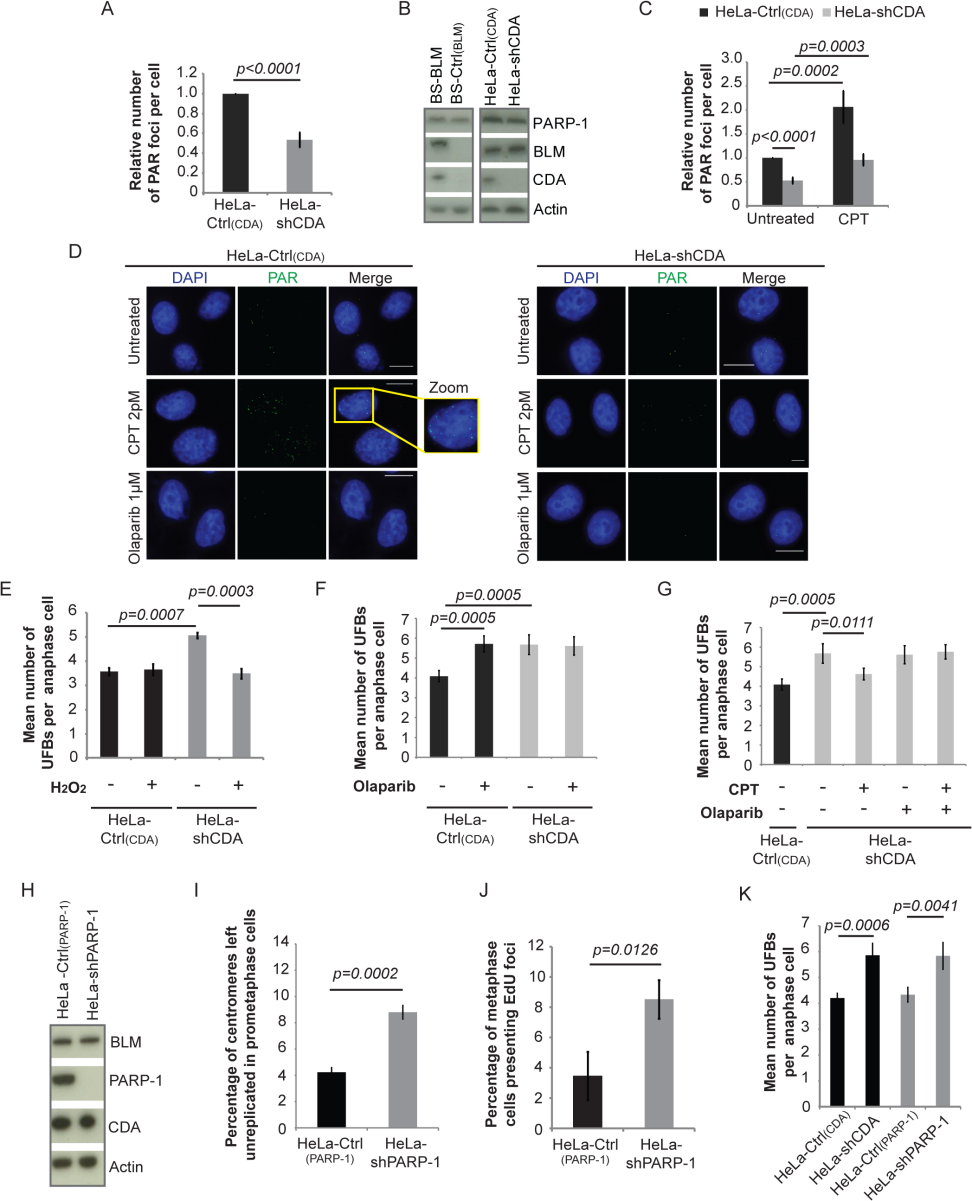
CDA deficiency is associated with a decrease in basal PARP-1 activity, leading to incomplete replication of DNA, promoting UFB formation. (A) Relative number of PAR foci in HeLa-Ctrl_(CDA)_ (black bar) and HeLa-shCDA (gray bar) cell lines. Error bars represent means ± SD from three independent experiments (> 680 cells per condition). (B) Abundance of PARP-1, BLM and CDA proteins, assessed by immunoblotting, in the indicated cell lines. (C) Relative number of PAR foci in HeLa-Ctrl_(CDA)_ (black bars) and HeLa-shCDA (gray bars) cells left untreated or treated with 2 pM CPT. Error bars represent means ± SD from five experiments (> 530 cells per condition). (D) Representative immunofluorescence images of HeLa-Ctrl_(CDA)_ (left panel) and HeLa-shCDA (right panel) interphase cells. Nuclei were visualized by DAPI staining (blue). PAR foci were stained with PAR antibody (in green). Boxed regions are enlarged. Scale bar: 5 μm. (E) Mean number of UFBs per anaphase cell, for HeLa-Ctrl_(CDA)_ (black bars) and HeLa-shCDA (gray bars) cell lines treated with 30 μM H_2_O_2_ (according to the same protocol as for CPT treatment, Fig 3A). Error bars represent means ± SD from three independent experiments (> 90 anaphase cells per condition). (F) Mean number of UFBs per anaphase cell, for the HeLa-Ctrl_(CDA)_ (black bars) and HeLa-shCDA (gray bars) cell lines left untreated or treated with 1 μM olaparib. Error bars represent means ± SD from five independent experiments (> 120 anaphase cells per condition). (G) Mean number of UFBs per anaphase cell, for the HeLa-Ctrl_(CDA)_ (black bars) and HeLa-shCDA (gray bars) cell lines left untreated or treated with 2pM CPT and/or 1 μM olaparib. Error bars represent means ± SD from five independent experiments (> 120 anaphase cells per condition). (H) Amounts of BLM, PARP-1, and CDA, assessed by immunoblotting, in the indicated cell lines. (I) Percentage of centromeres left unreplicated in HeLa-Ctrl_(PARP-1)_ (black bar) and HeLa-shPARP-1 (gray bar) prometaphase cells. Error bars represent means ± SD from three independent experiments (> 105 metaphase cells per condition) (J) Percentage of metaphase cells presenting EdU foci in HeLa-Ctrl_(PARP-1)_ (black bar) and HeLa-shPARP-1 (gray bar) cell lines. Error bars represent means ± SD for three independent experiments (> 115 metaphase cells were analyzed). (K) Mean number of UFBs per anaphase cell in the indicated cell lines. Error bars represent means ± SD from four independent experiments (> 105 anaphase cells per condition). Statistical significance was calculated with Student’s *t*-test.

We tested whether PARP-1 activity is compromised in CDA deficient cells. We assayed basal levels of PARylation by immunofluorescence microscopy and customized software for automatic counting of PAR foci. At least 500 cells per condition were counted for each experiment (S4A Fig and materials and methods). Basal PARylation levels were significantly lower in CDA-deficient HeLa and BS cells than in control cells (Fig 4A and S4C Fig). This was not due to a weaker PARP-1 expression in CDA-deficient cells (Fig 4B). There were twice as many cells without PAR foci and fewer cells with three or more foci in the absence than presence of CDA (S4B Fig). Therefore, basal PARP-1 activity is lower in the absence of CDA.

CPT treatment abolished the mitotic defects observed in CDA-deficient cells (Fig 2B and 2C), so we tested whether CPT treatment restored PARP-1 activity. CPT treatment activated PARP-1 in both CDA-deficient cells and control cells (Fig 4C and S4C Fig). The frequency of PAR foci (Fig 4C and 4D and S4C Fig) and numbers of UFBs (Fig 2B and Fig 2C) in CPT-treated CDA-deficient cells were similar to those in untreated control cells. We treated cells with H_2_O_2_, another PARP-1 activator [43]. The treatment of CDA-depleted cells with H_2_O_2_ increased PARylation (S4D Fig), and normalized the UFB frequency (Fig 4E). We assessed the dependence on PARP-1 activity of the suppressor effect of CPT, using the PARP-1 inhibitor olaparib [44]. Olaparib treatment (S4E Fig) reduced the frequency of PAR foci in both CDA-proficient and CDA-deficient cells, and abolished the effect of CPT on PARylation levels without changing CDA or PARP-1 levels (S4F and S4G Fig). Olaparib treatment also increased UFB frequency in CDA-expressing cells, but not in CDA-deficient cells (Fig 4F and S4H Fig). As expected, CPT treatment did not prevent supernumerary UFB formation in CDA-deficient cells treated with olaparib to inhibit PARP-1 (Fig 4G and S4H Fig).

These experiments show how CPT treatment alleviates the mitotic defects observed in the absence of CDA. There is a deficiency in the basal PARP-1 activity in CDA-deficient cells, and CPT treatment restores PARP-1 activity to levels similar to those in untreated control cells, rescuing the mitotic defects.

To further confirm these data, we used HeLa cells stably depleted of PARP-1 with a specific shRNA (HeLa-shPARP-1) [45, 46]. We checked that BLM and CDA levels were unaffected in these cells (Fig 4H). PARP-1-depleted cells displayed higher than control frequencies of unreplicated centromeres, metaphase cells presenting EdU foci, and UFBs, thereby mimicking CDA deficiency (Fig 4I–4K). As expected, these increases were not abolished by CPT treatment (S4I and S4J Fig).

Thus, regardless of how PAR synthesis was suppressed, through the inhibition or depletion of PARP-1, we observed an increase in the frequencies of unreplicated centromeres, EdU focus formation and UFBs. Moreover, these results also indicate that the effect of CPT in the prevention of excess UFB formation is strictly dependent on PARP-1 activity, with no additive effects of CDA deficiency and PARP-1 inhibition. CDA and PARP-1 must, therefore, act in the same pathway to prevent UFB formation. These results indicate that optimal PARP-1 activity is required for full centromere replication, which in turn prevents abnormal DNA synthesis and UFB formation during mitosis.

CDA deficiency leads to a slowing of the replication fork [25]. Decreases in PARP-1 activity due to CDA deficiency compromise the completion of DNA replication and lead to UFB formation. We therefore investigated whether PARP-1 depletion affected replication fork speed: PARP-1 depletion accelerated fork progression (S4K Fig), whereas CDA-deficiency slowed replication (S1H Fig). Thus, the completion of DNA replication, which prevents the formation of UFB-containing unreplicated DNA, is not directly related to the overall rate of fork progression.

### CDA deficiency leads to excess dCTP, lowering PARP-1 activity and promoting UFB formation

Our results indicated that long-term dU supplementation rescued the excess of UFBs (Fig 1E and S1J Fig), suggesting a possible influence of dUTP pool on PARP-1 activity. We therefore investigated whether CDA deficiency was associated with lower levels of cellular dUTP, as expected [27]. We detected no decrease in cellular dUTP levels in the absence of CDA (S5A Fig), suggesting that dU supplementation did not act by restoring the dUTP pool. These results prompted us to check whether culturing CDA-deficient cells in the presence of dU decreased UFB frequency through PARP-1 activation, rather than by mimicking CDA activity, as previously proposed [25]. We found that long-term dU treatment significantly increased the PAR signal (S5B Fig), indicating that the decrease in UFB frequency observed in dU-treated CDA-depleted cells was probably due to PARP-1 activation. These results suggest that the rescue by dU treatment involves a cellular metabolic process creating DNA lesions, similarly to CPT treatment. Indeed, we suggest that the addition of dU led to dUTP incorporation into DNA, activating the base excision repair pathway, and, thus, PARP-1, to ensure that dUTP is removed from the DNA [47, 48]. We conclude that the lower basal PARP-1 activity in CDA-deficient cells is probably not related to the dUTP pool, because it is unaffected in these cells.

**Fig 5:**
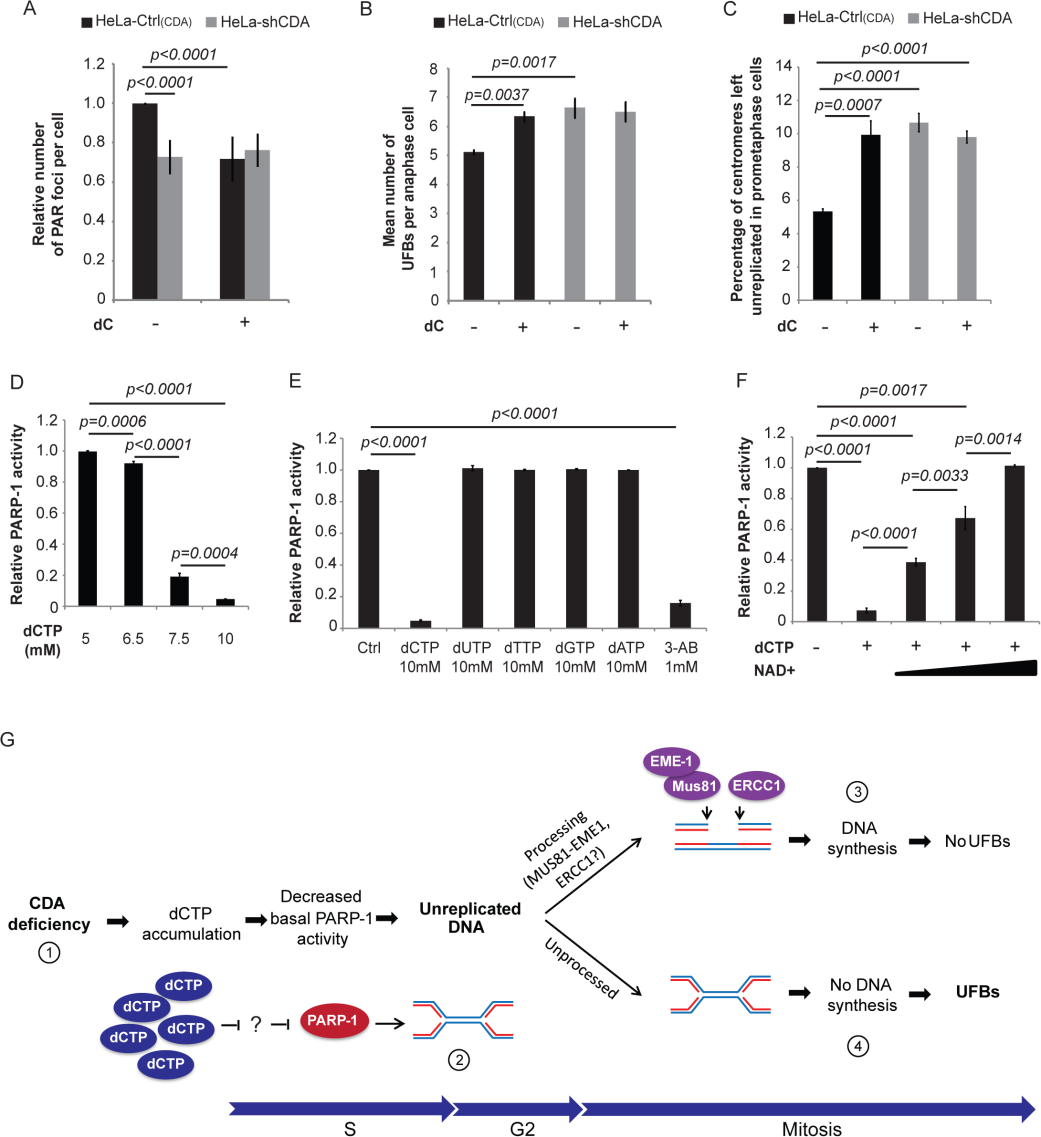
CDA deficiency leads to excess dCTP, inhibiting PARP-1 activity and leading to UFB formation. (A) Relative number of PAR foci in HeLa-Ctrl_(CDA)_ (black bars) and HeLa-shCDA (gray bars) cell lines treated with 1 mM dC. Error bars represent means ± SD for five independent experiments (> 1,200 cells per condition). (B) Mean number of UFBs per anaphase cell in HeLa-Ctrl_(CDA)_ (black bars) and HeLa-shCDA (gray bars) cell lines left untreated or treated with 1 mM dC. Error bars represent means ± SD for three independent experiments (> 95 anaphase cells per condition). (C) Percentage of centromeres left unreplicated in HeLa-Ctrl_(CDA)_ (black bars) and in HeLa-shCDA (gray bars) prometaphase cells left untreated or treated with 1 mM dC. Error bars represent means ± SD for three independent experiments (> 95 anaphase cells per condition). (D) *In vitro* analysis of PARP-1 activity in the presence of various amounts of dCTP. Error bars represent means ± SD from three independent experiments. Statistical significance was calculated with Student’s *t*-test. (E) *In vitro* analysis of PARP-1 activity in the presence of 1 mM 3-AB or 10 mM dCTP, dUTP, dTTP, dGTP, dATP. Error bars represent means ± SD from three independent experiments. (F) *In vitro* analysis of PARP-1 activity in the presence of 10 mM dCTP and various amounts of biotinylated NAD^+^ (20; 50; 100 μM). Error bars represent means ± SD from three independent experiments. Statistical significance was calculated with Student’s *t*-test. (G) (1) CDA deficiency leads to intracellular dCTP accumulation, decreasing basal PARP-1 activity. (2) The decrease in basal PARP-1 activity leads to an increase in unreplicated DNA sequences (preferentially at centromeres and CFS sites). (3) During late G2/early mitosis, some of this unreplicated DNA is probably processed by MUS81-EME1 and ERCC1 nucleases as previously suggested [36, 12], leading to DNA synthesis during metaphase (EdU foci) and preventing supernumerary UFB formation. (4) Some of the excess unreplicated DNA sequences are not processed by the nucleases, leading to supernumerary UFB formation.

We then investigated whether excess dCTP impaired PARP-1 activity, because dCTP accumulation is an established consequence of CDA deficiency (S1G Fig). The addition of dC to the culture medium decreased PARylation levels by 23% in CDA-expressing HeLa cells, and 18% in BS-BLM cells. Moreover, dC treatment during S phase (S5C Fig) increased UFB frequencies in these cells to levels similar to those in the corresponding CDA-deficient cells (Fig 5A and 5B and S5D and S5E Fig). The addition of dC did not affect PARylation levels or UFB frequency in CDA-deficient cells (Fig 5A and 5B and S5D and S5E Fig). The presence of dC also significantly increased the frequency of unreplicated centromeres in CDA-expressing HeLa cells (Fig 5C and S5F Fig).

These unexpected results led us to check whether the partial cellular inhibition of PARP-1 by dC treatment, which is known to induce dCTP accumulation [25], reflected PARP-1 inhibition by dCTP. We used an *in vitro* colorimetric PARP assay kit (Trevigen), with 3-aminobenzamide (3-AB) as a control PARP-1 inhibitor. The inhibition of PARP-1 activity by dCTP was dose-dependent, and was 95% at 10 mM dCTP (Fig 5D and 5E). Interestingly, 10 mM dATP, dGTP, dTTP or dUTP had no effect on PARP-1 activity (Fig 5E), indicating that the only dNTP to inhibit PARP-1 *in vitro* is dCTP. This inhibition was fully reversed by the addition of 100 μM NAD^+^ (NAD^+^ is the PARP-1 substrate used as a donor of ADP-ribose units for PARylation reactions [28]) indicating that dCTP is a poor inhibitor of PARP-1 *in vitro* (Fig 5F). Therefore, PARP-1 inhibition by dCTP *in vivo* is unlikely in the presence of the concentrations of dCTP and NAD^+^ found in cells (see the discussion).

Nevertheless, these analyses demonstrate that increasing the amount of intracellular dCTP by culturing cells in the presence of dC is sufficient to reduce PARP-1 activity.

## Discussion

We have discovered a novel mechanism by which the distortion of the cellular dNTP pool can affect genome stability. CDA deficiency, and thus excess dCTP, cause under-replication of some loci in the genome, leading to UFB formation. Unexpectedly, this incomplete DNA replication was found not to be due to the slowing of the replication fork induced by CDA deficiency. We show that the under-replication of “difficult-to-replicate” sites is a consequence of partial inhibition of the basal activity of PARP-1, likely due to the intracellular accumulation of dCTP. These findings reveal, unexpectedly, that PARP-1 is required for robust DNA replication and thus the avoidance of unreplicated DNA-associated UFBs. This work has broader implications for pathological situations associating higher replication stress and cancer susceptibility, such as Bloom syndrome.

### Excessive UFB formation in BS cells is a consequence of CDA deficiency and not BLM deficiency

We previously showed that CDA deficiency slows the progression of the replication fork and contributes to the high levels of SCEs in BS cells [25]. We report here that supernumerary UFBs in BS cells are entirely and solely due to CDA deficiency. In contrast, high chromatin bridge frequency results directly from BLM deficiency, and not from CDA deficiency. Our study demonstrates that these two types of sister-chromatid bridges have distinct genetic causes and presumably therefore different origins. This conclusion contrasts with a previous report suggesting that UFBs are derived from chromatin bridges through chromatin unraveling by PICH and BLM [49]. We also show that CDA deficiency makes a major contribution to several aspects of the cellular phenotype associated with Bloom syndrome. In view of this, all observations relating to or following BLM depletion should be interpreted with care: BLM depletion leads to CDA depletion [25, 26], and some of the cellular abnormalities observed may result from nucleotide pool disequilibrium associated with CDA deficiency, rather than BLM depletion *per se*.

### UFB-containing unreplicated DNA results from low basal PARP-1 activity and is not related to the rate of fork progression

The precise nature and structure of UFBs remains to be established. Our data indicate that both centromeric and CFS-associated UFBs likely contain unreplicated DNA, suggesting that, like CFS-associated UFBs, centromeric UFBs originate from unresolved replication intermediates. The frequency of mitotic DNA synthesis foci that co-localize with centromeres and CFS was abnormally high in CDA-deficient cells. The nature of these DNA synthesis events is still unclear, but these observations are consistent with centromeres and CFS being late-replicated loci in the absence of CDA, the duplication of which may be completed into mitosis. The persistence of mitotic DNA synthesis may allow the completion of DNA replication/repair of sites that were not fully replicated by the end of S phase; however, it is possible that only few sites are able to benefit from this mitotic processing [36, 12], such that many remain unreplicated, leading to UFB formation (Fig 5G). In support of this, the duplication of centromeres was impaired in the absence of CDA when cells enter mitosis.

CDA deficiency caused a lowering of basal PARP-1 activity. The reactivation of PARP-1 activity by exposing cells to genotoxic agents that activate PARP-1 was sufficient to restore the completion of DNA replication and suppress the formation of supernumerary UFBs. In addition, both depletion and chemical inhibition of PARP-1 resulted in partially replicated centromeres and under-replication-induced UFBs. These findings reveal a previously unsuspected role for PARP-1 in contributing to the robustness of DNA replication and preventing abnormal segregation of unreplicated DNA.

In contrast to CDA deficiency, stable down-regulation of PARP-1 accelerated replication fork progression. In previous studies, transient or chemical inhibition of PARP-1 did not affect replication fork speed [33, 50]. Similarly, while PARP-1 inhibition was reported to limit fork reversal upon genotoxic stress [33, 34, 51], the partial reduction in PARP-1 activity reported here in CDA-defective cells was associated with an increase in the rate of fork reversal, probably due to the observed increased in the frequency of discontinuities on the replication template [32]. The reasons for these discrepancies are unclear but could be related to the way PARP-1 activity was inhibited. Nonetheless, the completion of DNA replication, which prevents the formation of UFB-containing unreplicated DNA, is not directly related to the rate of fork progression.

Our analyses show that the deleterious effects of nucleotide pool disequilibrium on genome stability extend further than previously anticipated, and include inhibition of PARP-1 activity. PARP-1 defects may therefore be a source of endogenous replication stress. A decrease in basal PARP-1 activity of about 30% led to the formation of a number of supernumerary UFBs similar to that observed following total PARP-1 inhibition or depletion. This suggests that there is a critical basal threshold of PARP-1 activity for full replication of the genome before the cells enter mitosis. Although PARP-1 promoted the recovery of stalled replication forks through homologous recombination (HR) [52] inhibition of RAD51, and thereby of HR does not affect UFB frequency [15, 16]. Therefore, PARP-1 may also contribute to the completion of replication at AT-rich DNA sequences through a mechanism independent of HR.

Thousands of proteins, involved in diverse biological functions, are substrates of PARP-1 [39]. Most PARP-1 targets have been identified on the basis of responses to genotoxic stresses and basal PARylation levels of specific targets are difficult to determine [39]. In the absence of CDA, exposure of cells to genotoxic stress restored the activation of PARP-1 masking abnormal PARylation of individual substrates. Thus, further work is required to identify PARP-1 substrates that prevent UFB formation.

### The intracellular accumulation of dCTP inhibits PARP-1 activity

CDA deficiency leads to an excess dCTP. We report that increasing the intracellular dCTP pool *in vivo* by culturing cells in the presence of dC was sufficient to reduce PARP-1 activity, leading to the under-replication-induced formation of UFBs. We also showed that dCTP was the only dNTP that inhibited PARP-1 activity, although this inhibition was observed only at high dCTP concentrations (between 6.5 and 10 mM) and was fully reversed by 100 μM NAD^+^. The physiological intracellular concentrations of dCTP and NAD^+^ have been estimated to be about 30 μM and 1 mM, respectively [53, 54], and CDA-deficient cells contain about twice as much dCTP as control cells [25] (S1G Fig). As 100 μM NAD^+^ is sufficient to reverse the PARP-1 inhibition induced by 10 mM dCTP *in vitro*, it is unlikely that dCTP compete with NAD^+^ *in vivo*, to inhibit PARP-1. However, we cannot formally exclude the possibility that a particular environment within cells influences the local NAD^+^/dCTP ratio during replication, making it possible for dCTP to inhibit PARP-1 directly. The analysis of local concentrations of nucleotides at sites of DNA replication or repair remains difficult and this issue has been little explored, but such analyses in the future would undoubtedly shed light on new mechanisms.

In conclusion, our findings indicate that in CDA-deficient cells, the presence of excess intracellular dCTP caused a lowering of basal PARP-1 activity, impeding replication at some chromosomal loci, including centromeres and CFS, on entry into mitosis. Unreplicated DNA that is not processed by mitotic DNA synthesis results in the formation of supernumerary UFBs (Fig 5G).

## Materials and Methods

Detailed materials and methods are provided in *supporting information* (S1 Materials and Methods).

### Cell culture and treatments

Cell lines were cultured in DMEM supplemented with 10% FCS. BS-Ctrl_(BLM)_, BS-BLM cells, BS-Ctrl_(CDA)_ and BS-CDA were obtained and cultured as previously described [25].

The BS-BLM-CDA cell line was obtained by transfecting BS-BLM cells with a vector containing the full-length CDA cDNA (NM001785), with JetPEI reagent. After 48 h, selection was carried out with 0.2 μg.ml^− 1^ puromycin (Invivogen) and 500 μg.ml^− 1^ G418 (Euromedex). Individual colonies were isolated and maintained in culture with 0.1 μg.ml^− 1^ puromycin and 500 μg.ml^− 1^ G418.

HeLa-Ctrl_(CDA)_ and HeLa-shCDA cells were obtained by transfecting cells with an empty pGIPZ vector or with the same vector encoding a short hairpin RNA sequence directed against CDA (Open Biosystems, clone V3LHS_369299), respectively, with JetPEI reagent. After 48 h, transfectants were selected on 1–5 μg.ml^− 1^ puromycin (Invivogen). Individual colonies were isolated and cultured in medium containing 1 μg.ml^− 1^ puromycin.

HeLa-Ctrl_(PARP-1)_ and HeLa-shPARP-1 cells were cultured as previously described [46].

For siRNA transfection assays, 3×10^5^ HeLa cells or 8×10^5^ BS-Ctrl_(BLM)_, BS-BLM, BS-Ctrl_(CDA)_, BS-CDA, or BS-BLM-CDA cells were used to seed the wells of a six-well plate. Cells were transfected with an siRNA specific for BLM or CDA (ON-TARGETplus SMARTpool, Dharmacon) or negative control siRNAs (ON-TARGETplus siCONTROL Non Targeting Pool, Dharmacon; 100 nM final concentration) for 48 h for BLM, or twice successively, for a total of 120 h for CDA, in the presence of DharmaFECT 1 (Dharmacon).

Deoxyuridine (dU), deoxycytidine (dC), deoxyuridine triphosphate (dUTP), deoxycytidine triphosphate (dCTP), deoxyadenosine triphosphate (dATP), deoxyguanosine triphosphate (dGTP) and thymidine triphosphate (dTTP) were provided by Sigma Aldrich (D5412; D0779; D4001, D4635, D6500, D4010 and T0251 respectively); tetrahydrouridine (THU) was provided by Calbiochem (584222); camptothecin (CPT) was provided by Sigma Aldrich (C9911) and olaparib was provided by SelleckChem (S1060). THU and dU were added to the cell culture medium at a final concentration of 100 μM, for 96 h (2x48 h). Other drugs were added to the cell culture medium at the following concentrations: dC, 1 mM; H_2_O_2_, 30 μM; camptothecin, 2 pM; olaparib, 1 μM.

All cells were routinely checked for mycoplasma infection.

### Western blot analysis and antibodies

Cells were lysed in 8 M urea, 50 mM Tris HCl, pH 7.5 and 150 mM β-mercaptoethanol, sonicated and heated at 75°C for 10 minutes. Samples (equivalent of 2 x 10^5^ cells) were subjected to electrophoresis in NuPAGE Novex 4–12% Bis-Tris pre-cast gels (Life Technologies). The procedures used for gel electrophoresis and immunoblotting have been described elsewhere [16]. Primary and secondary antibodies were used at the following concentrations: rabbit anti-BLM antibody (1:5,000; ab2179 from Abcam); rabbit anti-CDA antibody (1:500; ab56053 from Abcam); rabbit anti-β-actin antibody (1:10,000; Sigma); rabbit anti-PARP-1 antibody (1:4,000; ALX-210-302 from Enzo Life Sciences); rabbit anti-Chk2 (1/500; 2662 from Cell Signaling; rabbit anti-Chk2 T68 (1/500; 2661 from Cell Signaling; rabbit anti-H2AX (1/500; 2595 from Cell Signaling); rabbit anti-H2AX S139 (1/500; 2577 from Cell Signaling); horseradish peroxidase-conjugated goat anti-rabbit IgG (1:5,000; Santa Cruz Biotechnology).

### Immunofluorescence microscopy

Immunofluorescence staining and analysis were performed as previously described [17]. Details of the protocol are provided in *SI Appendix*, *SI Materials and Methods*.

### Statistical analysis

At least three independent experiments were carried out to generate each dataset and the statistical significance of differences was calculated with Student’s *t*-test, Kruskal-Wallis tests or Mann and Whitney tests, as indicated in the figure legends. We distinguished true colocalization from the random colocalization of EdU foci and CREST or FANCD2 foci, by analyzing the images with the Image J macro “confined displacement algorithm” [37].

## Supporting Information

S1 Fig CDA prevents supernumerary UFB formation. (A) Mean number of UFBs per anaphase cell in the BS-Ctrl_(BLM)_, BS-BLM, BS-Ctrl_(CDA)_, and BS-CDA cell lines and the respective proportions of these UFBs associated with CREST (black bars, left panel) or FANCD2 (black bars, right panel). Error bars represent means ± SD from four independent experiments (> 120 anaphase cells per condition). (B-C) Percentages of anaphase cells presenting 0, 1, 2 or 3 or more UFBs in (B) BS-Ctrl_(BLM)_ and BS-BLM cell lines and in (C) BS-Ctrl_(CDA)_, and BS-CDA cell lines. Data are from five independent experiments (> 170 anaphase cells per condition). (D) Mean number of UFBs (black bars) and chromatin bridges (gray bars) per anaphase cell in BS-Ctrl_(CDA)_, BS-BLM-Ctrl and BS-BLM-CDA cell lines (left panel); BLM and CDA levels, assessed by immunoblotting (right panel). Errors bars represent means ± SD from three independent experiments (>120 anaphase cells per condition (UFBs) or > 70 anaphase cells per condition (chromatin bridges)). (E) BLM and CDA levels, assessed by immunoblotting, in BS-Ctrl_(BLM)_ and BS-BLM cell lines left untreated or treated with 100 μM dU or 100 μM THU. (F) CDA protein and mRNA were assayed by immunoblotting and by reverse transcription-quantitative PCR, respectively, in HeLa-Ctrl_(CDA)_ and HeLa-shCDA cells. Error bars represent means ± SD from three independent experiments. (G) HPLC analysis of the relative concentrations of dC and dCTP in HeLashCDA cells and HeLa-Ctrl_(CDA)_ cells. Error bars represent means ± SD from two independent experiments. (H) DNA combing analysis of replication fork velocity in HeLa-Ctrl_(CDA)_ (black bars) and HeLa-shCDA (gray bars) cells. Error bars represent the range of four independent experiments (>1400 replication tracts). Mann-Whitney tests were used to compare total numbers of DNA-positive tracts from the four experiments. (I) SCE frequencies in HeLa-Ctrl_(CDA)_ (black bars) and HeLa-shCDA (gray bars) cell lines. Error bars represent means ± SD from three independent experiments (> 1800 chromosomes analyzed for each condition). (J) Mean number of UFBs per anaphase cell in HeLa-Ctrl_(CDA)_ (black bars) and HeLa-shCDA (gray bars) cells left untreated or treated with 100 μM deoxyuridine (dU) or 100 μM tetrahydrouridine (THU). Statistical significance was calculated with Student’s t-test.
(TIF)

S2 Fig CDA deficiency does not promote global replication fork uncoupling but leads to the accumulation of ssDNA gaps at replication forks. (A) BLM and CDA abundance assayed by immunoblotting, in HeLa-Ctrl(CDA) and HeLa-shCDA cells left untreated or treated with 2pM CPT. (B) Cell cycle analysis of HeLa-Ctrl_(CDA)_ and HeLa-shCDA cells left untreated or treated with 2 pM CPT. (C) Representative EM images of replication forks in HeLa-Ctrl(CDA) and HeLa-shCDA cells. Black arrows indicate ssDNA gaps in parental or replicated duplexes, and white arrows indicate ssDNA gaps at the forks. The insets show magnified parts of the molecules, displaying ssDNA regions. Scale bars: 500 bp and 200 bp in the insets. (D) Table summarizing the size of the gaps at replication forks in HeLa-Ctrl_(CDA)_ and HeLa-shCDA cell lines left untreated or treated with 2 pM CPT. (E) Statistical analysis of the size of the gaps at the forks in HeLa- Ctrl_(CDA)_ and HeLa-shCDA cells left untreated or treated with 2 pM CPT. Whiskers indicate the minimum and maximum values in Kruskal-Wallis tests. No significant difference was observed between the four series. (F) Percentage of replication forks with 1 (black bars) or more than 1 (gray bars) ssDNA gap in HeLa-Ctrl_(CDA)_ and HeLa-shCDA cells left untreated or treated with 2 pM CPT. (G) Chk2 T68 and H2AX S139 levels, assessed by immunoblotting, in HeLa-Ctrl_(CDA)_ and HeLa-shCDA cells (left panel) and quantification of band intensity for Chk2 T68 and H2AX S139 relative to total protein (right panel). (H) Percentage fork reversal in HeLa-Ctrl_(CDA)_ and HeLa-shCDA cells left untreated or treated with 2 pM CPT. At least 50 replication forks were analyzed to quantify the percentage of replication forks with ssDNA gaps and the percentage of fork reversal.
(TIF)

S3 Fig CREST and FANCD2 associate with regions close to areas of mitotic DNA synthesis. Representative immunofluorescence deconvoluted z‐projection images of HeLa-Ctrl_(CDA)_ cells. DNA was visualized by DAPI staining (blue). EdU was stained with Alexa Fluor 555 (in magenta). Centromeres were stained with CREST serum (in green, upper panel) and CFS were stained by FANCD2 antibody (in green, lower panel). Boxed images are enlarged; yellow arrows indicate EdU foci and white arrows indicate CREST or FANCD2 foci.
(TIF)

S4 Fig Optimal PARP-1 activity is required for the full replication of centromeres and to prevent DNA synthesis and UFB formation during mitosis. (A) The number of PAR foci in each nucleus was determined by a customized macro using a semi-automated procedure. Briefly, each acquisition corresponding to DAPI and PAR staining was opened (Step 1). A user defined intensity value (one value for all experiments) was applied as a threshold (Step 2). The nucleus stack was smoothed using a median filter (radius 5), and a mask generated. This mask was transferred onto the focus stack so that only foci in nuclei were analyzed (Step 3). A top-hat filter was applied to this result to eliminate the local background, and facilitate the segmentation process based on application of a user-defined threshold value. Finally, the macro counted and characterized the foci (Step 4). At least 500 nuclei were analyzed for each condition. (B) Percentage of nuclei with various numbers of PAR foci in HeLa-Ctrl_(CDA)_ and HeLa-shCDA cells. Data are from three independent experiments (> 680 cells per condition). (C-D) Relative number of PAR foci in indicated cell lines left untreated or treated with 2 pM CPT (C) or with 30 μM H_2_O_2_ (D). Error bars represent means ± SD from (C) three independent experiments (a total of > 500 cells per condition) or (D) three independent experiments (> 360 cells per condition). (E) Schematic representation of 10 hours of treatment with 2 pM CPT and/or 1 μM olaparib during the cell cycle; only cells treated during the S and G2 phases were analyzed in anaphase. (F) Immunoblot assays of PARP-1 and CDA in HeLa-Ctrl_(CDA)_ and HeLa-shCDA cells left untreated or treated with 2 pM CPT and/or 1 μM olaparib. (G) Relative number of PAR foci in indicated cell lines left untreated or treated with 2 pM CPT and/or 1 μM olaparib. Error bars represent means ± SD from four independent experiments (> 500 cells per condition). (H) Mean number of UFBs per anaphase cell in BS-Ctrl_(BLM)_ (gray bars) and BS-BLM (black bars) cells left untreated or treated with 2pM CPT and/or 1 μM olaparib. Error bars represent means ± SD from three independent experiments (> 105 anaphase cells per condition). (I) Percentage of metaphase cells presenting EdU foci in HeLa-Ctrl_(PARP-1)_ (black bars) and HeLa-shPARP-1 (grey bars) cell lines treated with 2 pM CPT. Error bars represent means ± SD from three independent experiments (> 105 metaphase cells per condition). (J) Mean number of UFBs per anaphase cell in HeLa-Ctrl_(PARP-1)_ (black bars) and HeLa-shPARP-1 (gray bars) cells left untreated or treated with 2 pM CPT. Error bars represent means ± SD from three independent experiments (> 105 anaphase cells per condition). Statistical significance was calculated with Student’s t-test. (K) DNA combing analysis of replication fork velocity in HeLa-Ctrl_(PARP-1)_ (black bar) and HeLa-shPARP-1 (gray bar) cells. Error bars represent the range of three independent experiments (>730 replication tracts). Mann-Whitney statistical tests were used to study the results of all DNA-positive tracts analyzed in the three experiments.
(TIF)

S5 Fig CDA deficiency results in excess dCTP, inhibiting PARP-1 activity and leading to UFB formation. (A) HPLC analysis of the relative concentrations of dUTP in HeLa-shCDA cells and HeLa-Ctrl_(CDA)_ cells. Error bars represent means ± SD from three independent experiments. (B) Relative number of PAR foci in HeLa-Ctrl_(CDA)_ (black bars) and HeLa-shCDA (gray bars) cell lines treated with 100μM dU (96h). Error bars represent means ± SD for six independent experiments (> 850 cells per condition). (C) Schematic representation of dC treatment during the cell cycle; only cells treated during the S and G2 phases were analyzed in anaphase. (D) Relative number of PAR foci in BS-Ctrl_(BLM)_ (gray bars) and BS-BLM (black bars) cell lines left untreated or treated with 1 mM dC. Error bars represent means ± SD from three independent experiments (> 580 cells per condition). (E) Mean number of UFBs per anaphase cell, for BS-Ctrl_(BLM)_ (gray bars) and BS-BLM (black bars) cell lines left untreated or treated with 1 mM dC. Error bars represent means ± SD from three independent experiments (> 95 anaphase cells per condition). Student’s t-test was used to calculate the statistical significance of differences. (F) Percentage of chromosome 8 centromeres left unreplicated in HeLa-Ctrl_(CDA)_ (black bars) and in HeLa-shCDA (gray bars) metaphase cells left untreated or treated with 1 mM dC. Error bars represent means ± SD from three independent experiments (> 90 metaphase cells per condition). The statistical significance of differences was calculated with the Student’s t-test.
(TIF)

S1 Materials and Methods Detailed Materials and Methods. (DOC)
