# Supplementary figures and images for: Pyrimidine Pool Disequilibrium Induced by a Cytidine Deaminase Deficiency Inhibits PARP-1 Activity, Leading to the Under Replication of DNA
Source: PLoS Genet. 2015 Jul 16;11(7):e1005384. doi: 10.1371/journal.pgen.1005384 (PMC4504519; doi:10.1371/journal.pgen.1005384)

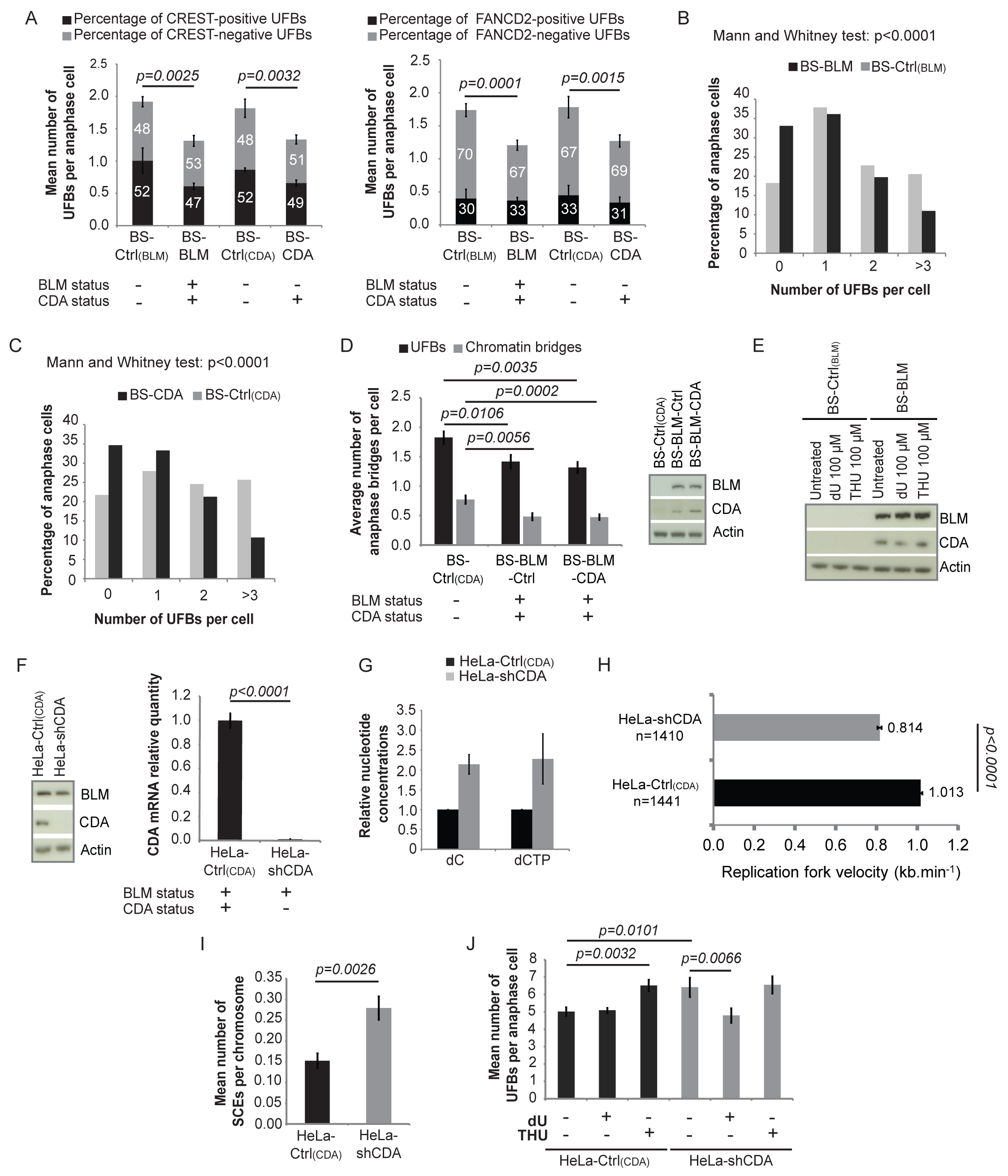

Supplement: S1 Fig — (A) Mean number of UFBs per anaphase cell in the BS-Ctrl(BLM), BS-BLM, BS-Ctrl(CDA), and BS-CDA cell lines and the respective proportions of these UFBs associated with CREST (black bars, left panel) or FANCD2 (black bars, right panel). Error bars represent means ± SD from four independent experiments (> 120 anaphase cells per condition). (B-C) Percentages of anaphase cells presenting 0, 1, 2 or 3 or more UFBs in (B) BS-Ctrl(BLM) and BS-BLM cell lines and in (C) BS-Ctrl(CDA), and BS-CDA cell lines. Data are from five independent experiments (> 170 anaphase cells per condition). (D) Mean number of UFBs (black bars) and chromatin bridges (gray bars) per anaphase cell in BS-Ctrl(CDA), BS-BLM-Ctrl and BS-BLM-CDA cell lines (left panel); BLM and CDA levels, assessed by immunoblotting (right panel). Errors bars represent means ± SD from three independent experiments (>120 anaphase cells per condition (UFBs) or > 70 anaphase cells per condition (chromatin bridges)). (E) BLM and CDA levels, assessed by immunoblotting, in BS-Ctrl(BLM) and BS-BLM cell lines left untreated or treated with 100 μM dU or 100 μM THU. (F) CDA protein and mRNA were assayed by immunoblotting and by reverse transcription-quantitative PCR, respectively, in HeLa-Ctrl(CDA) and HeLa-shCDA cells. Error bars represent means ± SD from three independent experiments. (G) HPLC analysis of the relative concentrations of dC and dCTP in HeLashCDA cells and HeLa-Ctrl(CDA) cells. Error bars represent means ± SD from two independent experiments. (H) DNA combing analysis of replication fork velocity in HeLa-Ctrl(CDA) (black bars) and HeLa-shCDA (gray bars) cells. Error bars represent the range of four independent experiments (>1400 replication tracts). Mann-Whitney tests were used to compare total numbers of DNA-positive tracts from the four experiments. (I) SCE frequencies in HeLa-Ctrl(CDA) (black bars) and HeLa-shCDA (gray bars) cell lines. Error bars represent means ± SD from three independent experiments (> 1800 ch [file pgen.1005384.s001.tif]

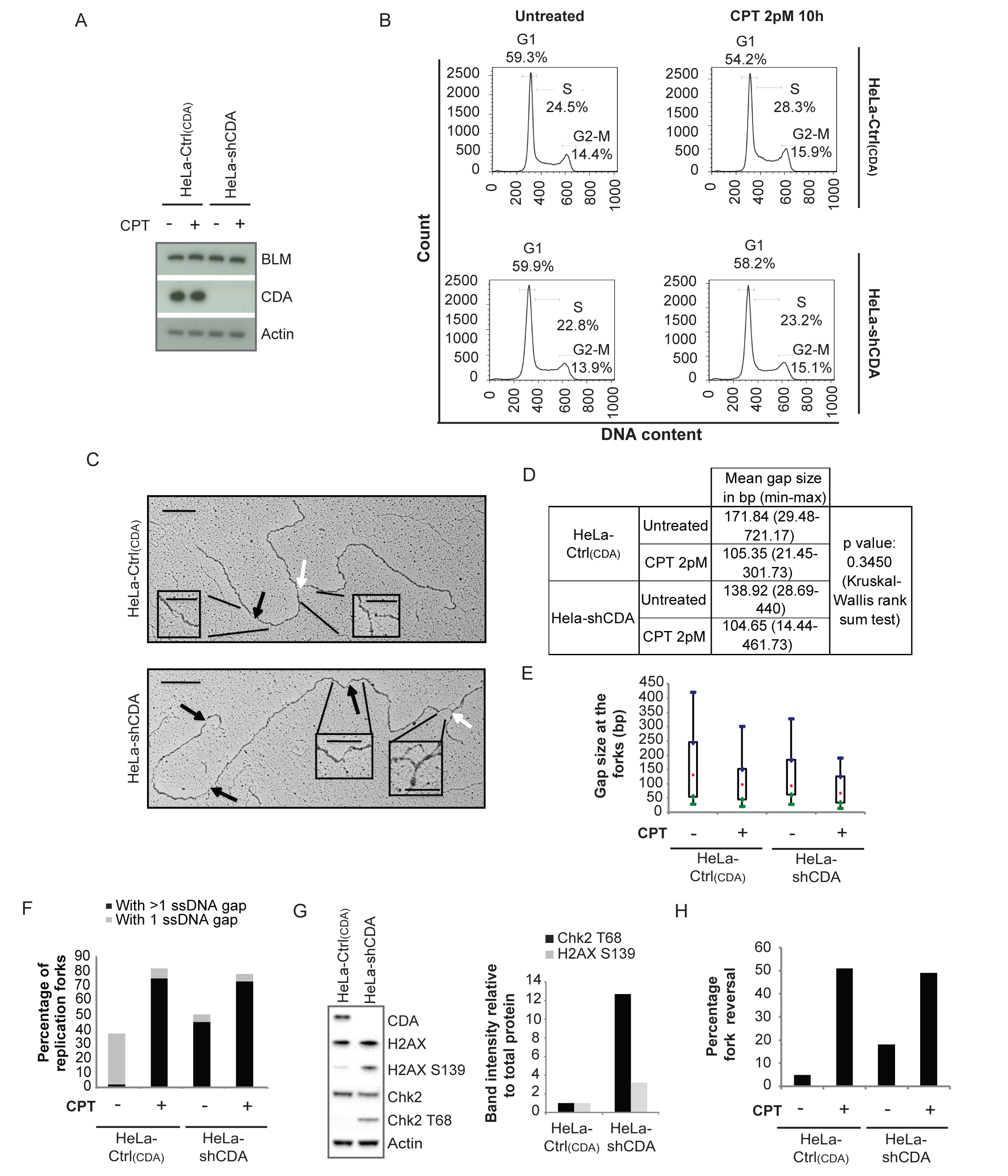

Supplement: S2 Fig — (A) BLM and CDA abundance assayed by immunoblotting, in HeLa-Ctrl(CDA) and HeLa-shCDA cells left untreated or treated with 2pM CPT. (B) Cell cycle analysis of HeLa-Ctrl(CDA) and HeLa-shCDA cells left untreated or treated with 2 pM CPT. (C) Representative EM images of replication forks in HeLa-Ctrl(CDA) and HeLa-shCDA cells. Black arrows indicate ssDNA gaps in parental or replicated duplexes, and white arrows indicate ssDNA gaps at the forks. The insets show magnified parts of the molecules, displaying ssDNA regions. Scale bars: 500 bp and 200 bp in the insets. (D) Table summarizing the size of the gaps at replication forks in HeLa-Ctrl(CDA) and HeLa-shCDA cell lines left untreated or treated with 2 pM CPT. (E) Statistical analysis of the size of the gaps at the forks in HeLa- Ctrl(CDA) and HeLa-shCDA cells left untreated or treated with 2 pM CPT. Whiskers indicate the minimum and maximum values in Kruskal-Wallis tests. No significant difference was observed between the four series. (F) Percentage of replication forks with 1 (black bars) or more than 1 (gray bars) ssDNA gap in HeLa-Ctrl(CDA) and HeLa-shCDA cells left untreated or treated with 2 pM CPT. (G) Chk2 T68 and H2AX S139 levels, assessed by immunoblotting, in HeLa-Ctrl(CDA) and HeLa-shCDA cells (left panel) and quantification of band intensity for Chk2 T68 and H2AX S139 relative to total protein (right panel). (H) Percentage fork reversal in HeLa-Ctrl(CDA) and HeLa-shCDA cells left untreated or treated with 2 pM CPT. At least 50 replication forks were analyzed to quantify the percentage of replication forks with ssDNA gaps and the percentage of fork reversal. (TIF) [file pgen.1005384.s002.tif]

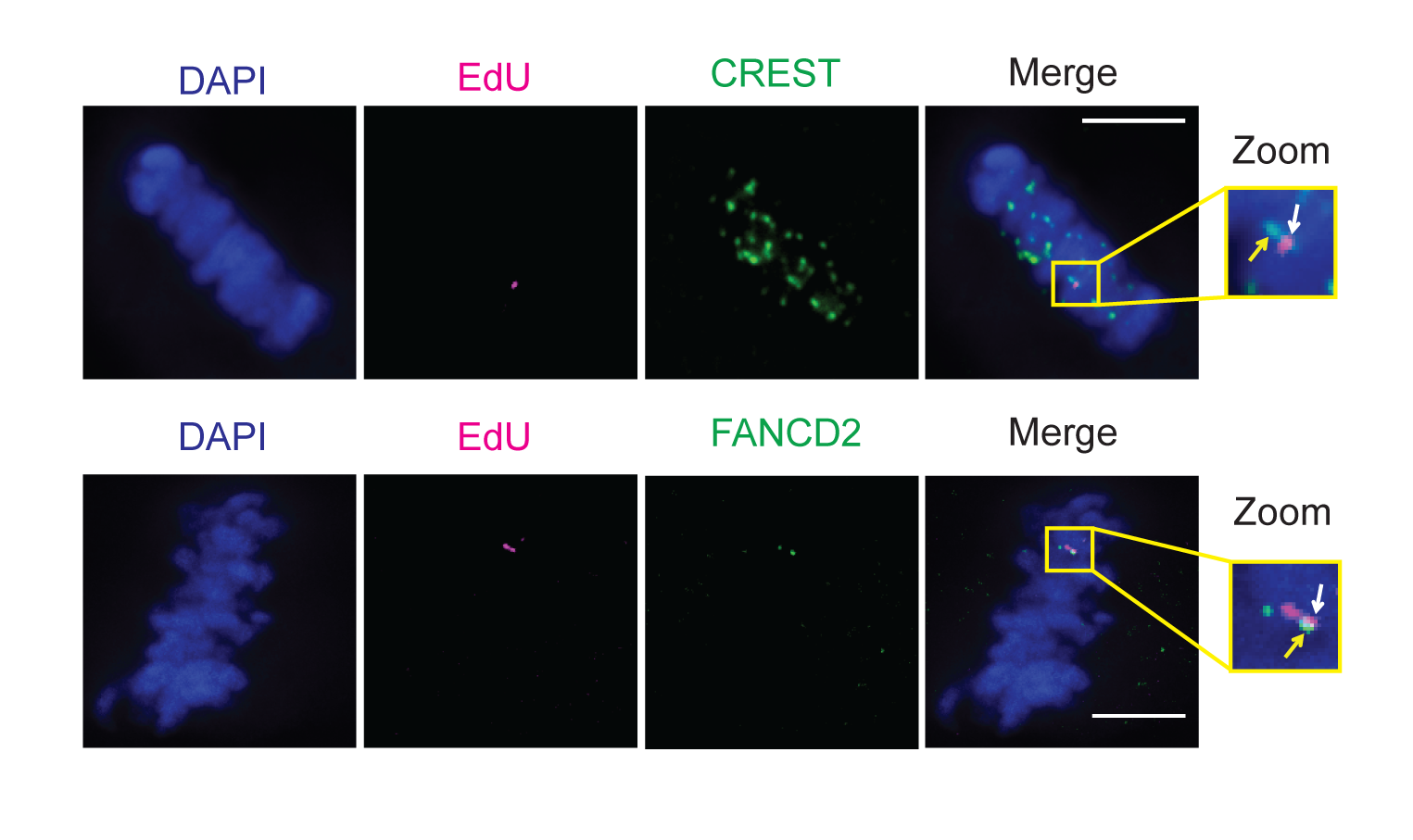

Supplement: S3 Fig — Representative immunofluorescence deconvoluted z‐projection images of HeLa-Ctrl(CDA) cells. DNA was visualized by DAPI staining (blue). EdU was stained with Alexa Fluor 555 (in magenta). Centromeres were stained with CREST serum (in green, upper panel) and CFS were stained by FANCD2 antibody (in green, lower panel). Boxed images are enlarged; yellow arrows indicate EdU foci and white arrows indicate CREST or FANCD2 foci. (TIF) [file pgen.1005384.s003.tif]

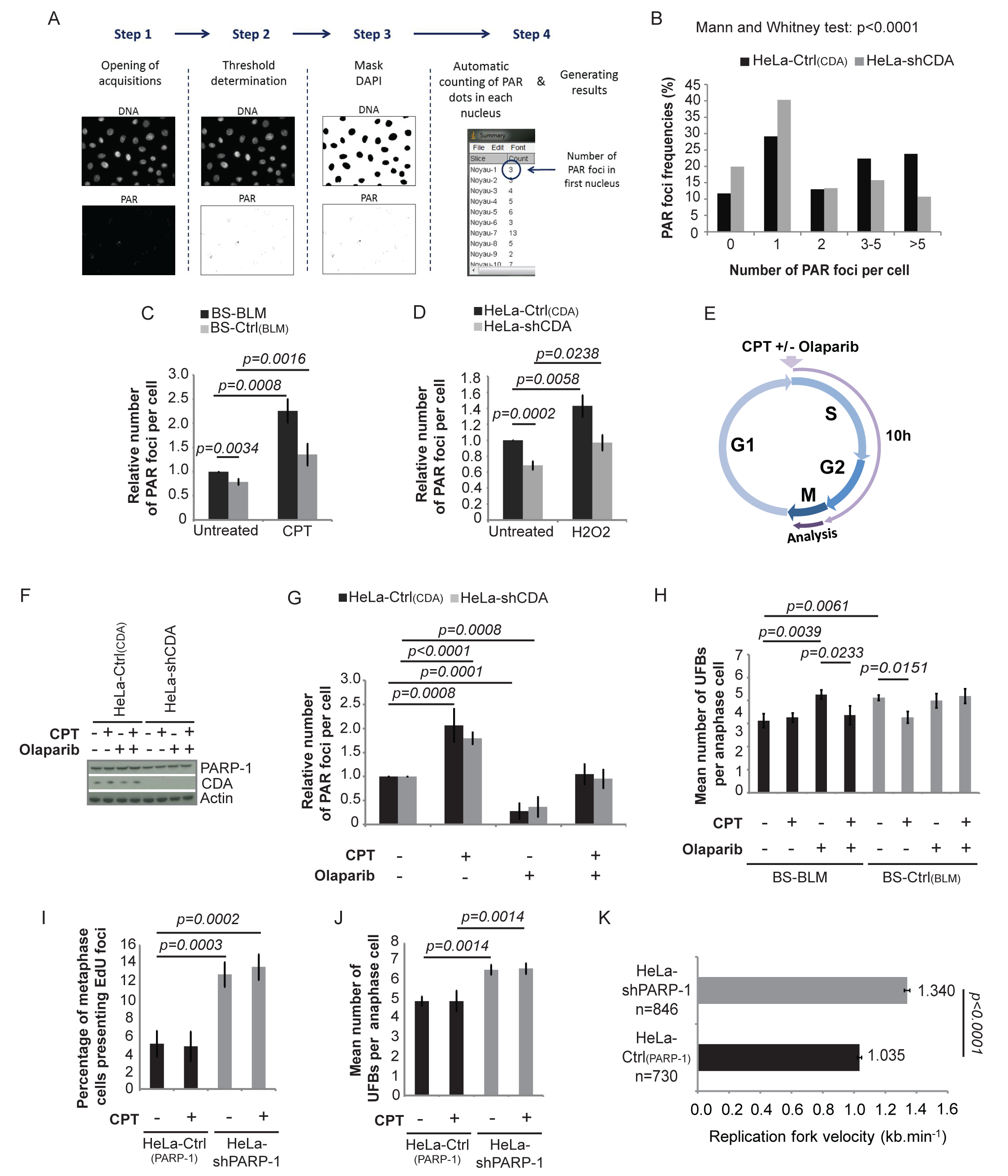

Supplement: S4 Fig — (A) The number of PAR foci in each nucleus was determined by a customized macro using a semi-automated procedure. Briefly, each acquisition corresponding to DAPI and PAR staining was opened (Step 1). A user defined intensity value (one value for all experiments) was applied as a threshold (Step 2). The nucleus stack was smoothed using a median filter (radius 5), and a mask generated. This mask was transferred onto the focus stack so that only foci in nuclei were analyzed (Step 3). A top-hat filter was applied to this result to eliminate the local background, and facilitate the segmentation process based on application of a user-defined threshold value. Finally, the macro counted and characterized the foci (Step 4). At least 500 nuclei were analyzed for each condition. (B) Percentage of nuclei with various numbers of PAR foci in HeLa-Ctrl(CDA) and HeLa-shCDA cells. Data are from three independent experiments (> 680 cells per condition). (C-D) Relative number of PAR foci in indicated cell lines left untreated or treated with 2 pM CPT (C) or with 30 μM H2O2 (D). Error bars represent means ± SD from (C) three independent experiments (a total of > 500 cells per condition) or (D) three independent experiments (> 360 cells per condition). (E) Schematic representation of 10 hours of treatment with 2 pM CPT and/or 1 μM olaparib during the cell cycle; only cells treated during the S and G2 phases were analyzed in anaphase. (F) Immunoblot assays of PARP-1 and CDA in HeLa-Ctrl(CDA) and HeLa-shCDA cells left untreated or treated with 2 pM CPT and/or 1 μM olaparib. (G) Relative number of PAR foci in indicated cell lines left untreated or treated with 2 pM CPT and/or 1 μM olaparib. Error bars represent means ± SD from four independent experiments (> 500 cells per condition). (H) Mean number of UFBs per anaphase cell in BS-Ctrl(BLM) (gray bars) and BS-BLM (black bars) cells left untreated or treated with 2pM CPT and/or 1 μM olaparib. Error bars represent means ± SD from three indep [file pgen.1005384.s004.tif]

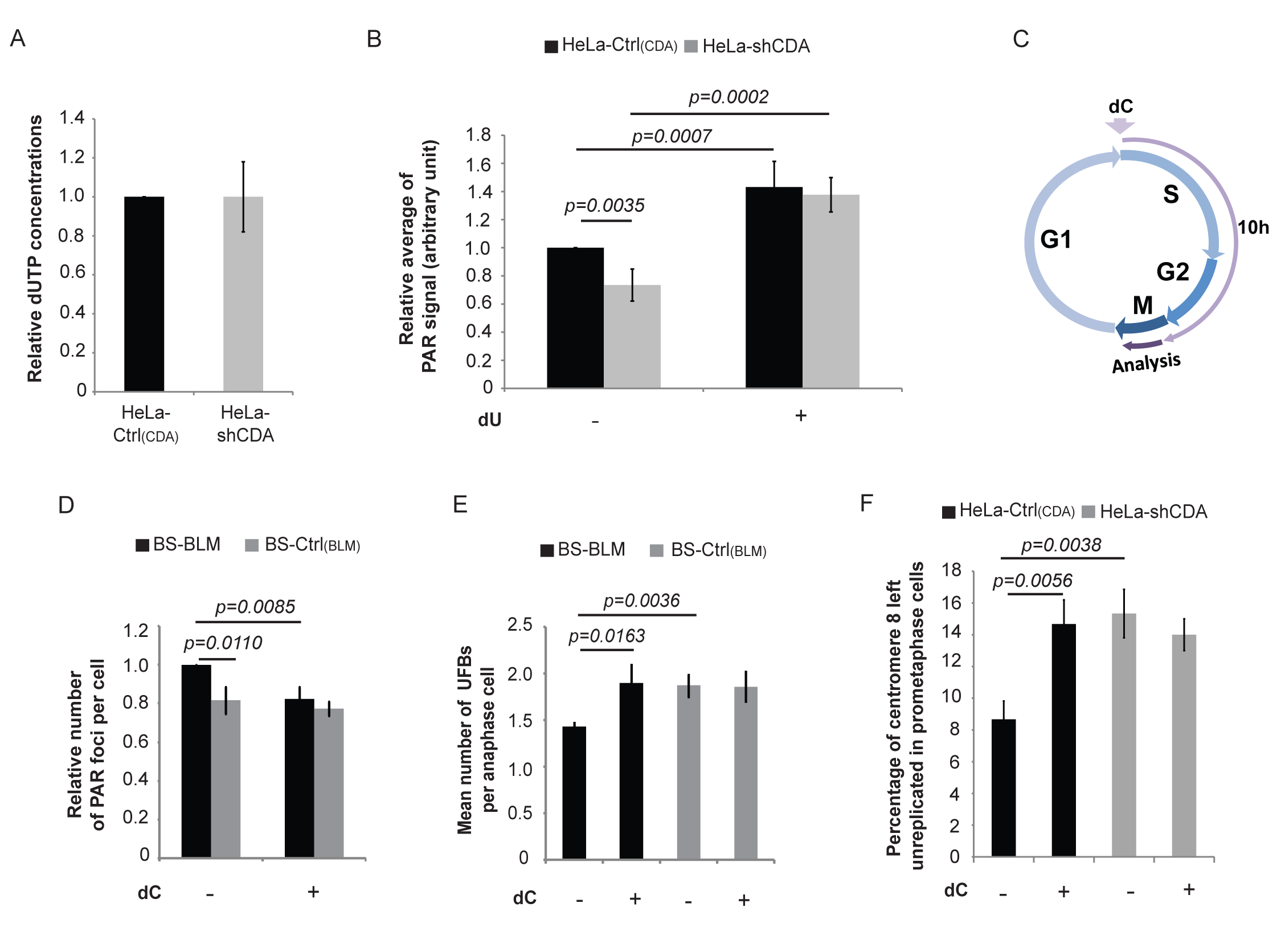

Supplement: S5 Fig — (A) HPLC analysis of the relative concentrations of dUTP in HeLa-shCDA cells and HeLa-Ctrl(CDA) cells. Error bars represent means ± SD from three independent experiments. (B) Relative number of PAR foci in HeLa-Ctrl(CDA) (black bars) and HeLa-shCDA (gray bars) cell lines treated with 100μM dU (96h). Error bars represent means ± SD for six independent experiments (> 850 cells per condition). (C) Schematic representation of dC treatment during the cell cycle; only cells treated during the S and G2 phases were analyzed in anaphase. (D) Relative number of PAR foci in BS-Ctrl(BLM) (gray bars) and BS-BLM (black bars) cell lines left untreated or treated with 1 mM dC. Error bars represent means ± SD from three independent experiments (> 580 cells per condition). (E) Mean number of UFBs per anaphase cell, for BS-Ctrl(BLM) (gray bars) and BS-BLM (black bars) cell lines left untreated or treated with 1 mM dC. Error bars represent means ± SD from three independent experiments (> 95 anaphase cells per condition). Student’s t-test was used to calculate the statistical significance of differences. (F) Percentage of chromosome 8 centromeres left unreplicated in HeLa-Ctrl(CDA) (black bars) and in HeLa-shCDA (gray bars) metaphase cells left untreated or treated with 1 mM dC. Error bars represent means ± SD from three independent experiments (> 90 metaphase cells per condition). The statistical significance of differences was calculated with the Student’s t-test. (TIF) [file pgen.1005384.s005.tif]
