# Supplementary material for: Pyrimidine Pool Disequilibrium Induced by a Cytidine Deaminase Deficiency Inhibits PARP-1 Activity, Leading to the Under Replication of DNA
Source: PLoS Genet. 2015 Jul 16;11(7):e1005384. doi: 10.1371/journal.pgen.1005384 (PMC4504519; doi:10.1371/journal.pgen.1005384)
Supplement: S1 Materials and Methods — (DOC) [file pgen.1005384.s006.doc]

**SUPPORTING INFORMATION**

**for**

**pyrimidine pool disequilibrium induced by A cytidine deaminase deficiency inhibits PARP-1 activity, leading to the under-REplication of DNA**

Simon Gemble^a,b^, Akshay Ahuja^c^, Géraldine Buhagiar-Labarchède^a,b^, Rosine Onclercq-Delic^a,b^, Julien Dairou^d^, Denis S.F. Biard^e^, Sarah Lambert^a,b^, Massimo Lopes^c^ and Mounira Amor-Guéret^a,b,1^

^a^Institut Curie, Centre de Recherche, Orsay, France

^b^CNRS UMR 3348, Stress Génotoxiques et Cancer, Centre Universitaire, Bât. 110. 91405, Orsay, France

^c^Institute of Molecular Cancer Research, University of Zurich, Zurich, Switzerland

^d^Université Paris Diderot, Sorbonne Paris Cité, Unité de Biologie Fonctionnelle et Adaptative (BFA) UMR 8251 CNRS, Plateforme Bioprofiler Bâtiment Buffon, 346A Case 7073, 75205 PARIS Cedex 13, France

^e^CEA, DSV, iMETI, SEPIA, 18, route du Panorama. Bât. 60, BP6, 92265 Fontenay-aux-Roses Cedex, France

^1^To whom correspondence should be addressed. E-mail: [**mounira.amor@curie.fr**](https://mail.curie.fr/owa/redir.aspx?C=wUfpjw7VB0q3Htgv5qOu3bz8cH7xrNEIJunxWYdSzAxDGt7PpyMRA4xp7B3oSp8dbva8SmhmN_w.&URL=mailto%3Amounira.amor@curie.fr)

*Keywords*: Bloom syndrome; Cytidine deaminase; Nucleotide pool imbalance; PARP-1; Under-replicated DNA; Ultrafine anaphase bridges

**1. SI Materials and Methods:**

**Immunofluorescence microscopy.** Immunofluorescence staining and analysis were performed as previously described (1). The drugs indicated were included in cell culture media for 3 or 10 h. Primary and secondary antibodies were used at the following concentrations: rabbit anti-PICH antibody (1/150; H00054821-D01P from Abnova); mouse anti-PICH antibody (1/400; H00054821-M01 from Abnova); human anti-CREST antibody (1/100; 15-234-0001 from Antibodies Inc); rabbit anti-FANCD2 antibody (1/200; NB100-182 from Novus Biologicals); goat anti-rabbit Alexa Fluor 555 (1/500; A21429 from Life Technologies); goat anti-mouse Alexa Fluor 555 (1/500; A21050 from Life Technologies); goat anti-rabbit Alexa Fluor 633 (1/500; A21070 from Life Technologies); goat anti-human Alexa Fluor 633 (1/500; A21091 from Life Technologies). Cell images were acquired with a 3-D deconvolution imaging system consisting of a Leica DM RXA microscope equipped with a piezoelectric translator (PIFOC; PI) placed at the base of a 63x PlanApo N.A. 1.4 objective, and a CoolSNAP HQ interline CCD camera (Photometrics). Stacks of conventional fluorescence images were collected automatically at a Z-distance of 0.2 mm (Metamorph software; Molecular Devices). Images are presented as maximum intensity projections, generated with ImageJ software, from stacks deconvolved with an extension of Metamorph software (2).

**Poly(ADP)-ribose immunofluorescence**. We used 4×10^5^ HeLa cells or 8×10^5^ BS-Ctrl_(BLM)_ or BS-BLM cells to seed the wells of a six-well plate. The cells were left untreated or were treated for 20 minutes with CPT, olaparib or H_2_O_2_, or for 1 h with dC. They were then washed in cold PBS on ice and fixed by incubation in a 1:1 (vol/vol) mixture of methanol and acetone for 10 minutes on ice. After three washes in PBS-Tween (0.05%), cells were incubated overnight at 4°C with a mouse anti-PAR antibody (1/500; generously provided by Valérie Schreiber (3)). The cells were washed three times with PBS-Tween (0.05%) and incubated with a goat anti-mouse Alexa Fluor 555-conjugated antibody (1/500; A21050 from Life Technologies) for 2 h. After two washes in PBS-Tween (0.05%), cells were mounted on slides with Prolong Gold with DAPI (P36931 from Life Technologies). Cell images were acquired with a 3-D deconvolution imaging system consisting of a Leica DM RXA microscope equipped with a piezoelectric translator (PIFOC; PI) placed at the base of a 63x PlanApo N.A. 1.4 objective, and a CoolSNAP HQ interline CCD camera (Photometrics). Stacks of conventional fluorescence images were collected automatically at a Z-distance of 0.2 mm (Metamorph software; Molecular Devices). Images are presented as maximum intensity projections, generated with ImageJ software, from stacks deconvolved with an extension of Metamorph software (2). PAR foci per nucleus were counted by a customized macro using a semi-automated procedure, as follows: the nucleus stack was first smoothed using a median filter (radius 5), the user defined an intensity value as a threshold (one value for all experiments); a mask was then generated and transferred onto the stack of foci so that only foci in nuclei were analyzed. A top-hat filter was applied to the result to eliminate local background and facilitate the segmentation process, based on a simple threshold (user defined value). Finally, the macro counted and characterized foci. At least 500 nuclei were analyzed for each condition.

**FISH Analysis.** FISH was performed with the CEP-8 probe (Cen-8) from Abbott Vysis according to the manufacturer’s instructions and as previously described (1).

**HPLC analysis.** We separated and quantified dC and dCTP as previously described (4, 5). Protein was extracted by incubation with 6% TCA and centrifugation for 5 minutes (12000 x *g*), and the resulting supernatants were neutralized with 2 M K_2_CO_3_. The samples were then treated on Strata X solid-phase extraction (SPE) cartridges, according to the manufacturer's instructions (Phenomenex). The resulting eluate was then injected, the same day, into the HPLC system (Shimadzu HPLC system interfaced with LabSolution software). Samples were injected onto a 250 x 4.1 mm PRP-1 column (Hamilton) and separated with buffer A for 8 min, followed by a linear gradient of buffer B from 0 to 75% over 62 minutes in buffer A, at a flow rate of 1 ml/min. Buffer A was 20 mM triethylammonium hydrogen carbonate and buffer B was 20 mM triethylammonium hydrogen carbonate in 20% methanol. A diode array detector (PDA) was used for detection. The LC Solution workstation chromatography manager was used to pilot the HPLC instrument and to process the data. The products were monitored spectrophotometrically at 254 and 260 nm and quantified by integration of the peak absorbance area, with a calibration curve established with various known concentrations of dC and dCTP.

**Electron microscopy analysis of genomic DNA in mammalian cells.** *In vivo* psoralen cross-linking, the isolation of total genomic DNA, enrichment in replication intermediates and their EM visualization were carried out as previously described (6, 7). Briefly, cells were harvested, and genomic DNA was crosslinked *in situ* by two rounds of incubation in 10 μM 4,5',8-trimethylpsoralen and two minutes of irradiation with UV light at a wavelength of 366 nm. Cells were lysed and genomic DNA was isolated from the nuclei by proteinase K digestion and phenol-chloroform extraction. The purified DNA was digested with *Pvu*II and enriched in replication intermediates by passage through a BND cellulose column. EM samples were prepared by spreading the DNA onto carbon-coated grids, and visualized by platinum rotary shadowing. Images were acquired with a Philips CM 100 microscope. The lengths of gaps were estimate by comparison to a plasmid reference: the plasmid was 8240 bp and measured 3530 nm when evaluated by ImageJ on a .dm3 image (Gatan Digital Micrograph software); thus, 8240 bp corresponded to 3530 nm, and consequently 1 nm = 2.3 bp.

**RT-qPCR.** RT-qPCR analysis was performed as previously described (8). Briefly, total RNA was extracted with the RNeasy Mini kit (Qiagen), ans subjected to a DNAse digestion step. The *A*_260_/*A*_280_ ratio was measured and the integrity of the RNA was assessed by gel electrophoresis. We synthesized cDNAs with 250 ng of random hexamers (Life Technologies), 2 µg of RNA and the GoScript reverse transcriptase (Promega). Quantitative PCR experiments were performed in accordance with MIQE Guidelines. Amplification mixtures contained the cDNA template (1/100 dilution), 1 × SYBR Green Supermix (BioRad) and 300 nM forward and reverse primers. Amplification was performed with the CFX96 detection system (BioRad). The relative values for CDA cDNA were normalized against those for two reference genes (*RPL32* and *HMBS*) chosen on the basis of their low M-values. The primer sequences for *RPL32* and *HMBS* have been described elsewhere (8). The sequences of the CDA primers used were: primer 1 (5′-CCCCTACAGTCACTTTCCTG-3′) and primer 2 (5′-CGGGTAGCAGGCATTTTCTA-3′).

**EdU staining.** EdU incorporation into DNA was visualized with the Click-it EdU imaging kit (C10338 from Life Technologies), according to the manufacturer’s instructions. EdU was used at a concentration of 10 µM for the indicated time. Cells were incubated with the Click-it reaction cocktail for 15 minutes. Cell images were acquired with a 3-D deconvolution imaging system consisting of a Leica DM RXA microscope equipped with a piezoelectric translator (PIFOC; PI) placed at the base of a 63x PlanApo N.A. 1.4 objective, and a CoolSNAP HQ interline CCD camera (Photometrics). Stacks of conventional fluorescence images were collected automatically at a Z-distance of 0.2 mm (Metamorph software; Molecular Devices). Images are presented as maximum intensity projections generated with ImageJ software, from stacks deconvolved with an extension of Metamorph software.

**Colorimetric PARP assay kit for candidate inhibitor screening:** PARP-1 inhibition by dCTP was determined with the HT universal colorimetric PARP assay kit, with histone-coated strip wells (4677-096-K from Trevigen), according to the manufacturer’s instructions. In the experiment reported in Figure 5F, biotinylated NAD^+^ (4670-500-01 from Trevigen) was added at the A5 step with the PARP cocktail. Absorbance was measured at 450 nm with a Victor 3 Multilabel Counter plate reader (Perkin Elmer).

**Sister chromatid exchange assay.** Cells were transferred to slides and cultured in the presence of 10 µM 5-bromodeoxyuridine (BrdU) (Sigma) for two divisions. After 40 h, colchicine (Sigma) was added (0.1 µg/ml) and the cells were incubated for 1 h in a hypotonic solution (1:5 (vol/vol) FCS (Fetal calf Serum) in distilled water) and the fixed by incubation with a 3:1 (vol/vol) mixture of methanol and acetic acid. They were then stained by incubation with 10 µg/ml Hoechst 33258 (Sigma) in distilled water for 20 minutes, rinsed with 2×SSC (Euromedex), exposed to ultraviolet light at a wavelength of 365 nm, at a distance of 10 cm for 105 minutes, rinsed in water, stained by incubation with 2% Giemsa solution (VWR) for 16 minutes, rinsed in water, dried and mounted in EUKITT (Sigma). Chromosomes were observed with a Leica DMRB microscope at ×100 magnification. Metaphases were captured with a SONY DXC 930 P camera and SCEs were analyzed.

**DNA molecular combing.** Asynchronous populations of cells were labeled by incubation for 20 minutes with 100 µM IdU (Sigma), washed with medium at 37°C and then labeled by incubation for 20 minutes with 100 µM CldU (Sigma). The DNA solution was prepared as previously described (9), and DNA was combed onto silane-treated coverslips (GenomicVision) with a combing apparatus (Genomic Vision). Coverslips with combed DNA were baked overnight at 60°C and incubated in 0.5 M NaOH and 1 M NaCl for 15 minutes, with gentle shaking, to denature the DNA. The coverslips were washed several times in PBS and dried by successive incubations, for 5 minutes each, in 70, 90 and 100% ethanol. They were then incubated with the primary antibodies. All antibodies were diluted in BlockAid (Life Technologies). Coverslips were incubated at room temperature, first with 1/5 mouse anti-BrdU antibody (BD Biosciences) and 1/25 rat anti-CldU antibody (AbDserotec) for 1 h, then with 1/25 goat anti-mouse Alexa Fluor 488 and 1/25 goat anti-rat Alexa Fluor 555 antibodies (Life Technologies) for 40 minutes, followed by 1/50 mouse anti-ssDNA antibody (Millipore MAB-3034) for 40 minutes, 1/25 rabbit anti-mouse Alexa Fluor 350 antibody (Life Technologies) for 30 minutes and, finally, with 1/25 goat anti-rabbit 350 Alexa Fluor antibody (Life Technologies) for 30 minutes. Between incubations, coverslips were washed three times, for 5 minutes each, in PBS. Coverslips were mounted in Prolong Gold antifade reagent (Life Technologies). Images were acquired with a Leica DM RXA microscope equipped with a motorized XY stage, using a ×40 PlanApo N.A. 1.25 objective and a CoolSNAP HQ interline CCD camera (Photometrics). For each slide, a mosaic of 10×10 partly overlapping images was collected with a Metamorph software (Molecular Devices) routine developed in-house. Image collections were assembled into a mosaic with the ‘Stitching 2D/3D’ plugin32 (available from http://fly.mpi-cbg.de/~preibisch/software.html) for ImageJ software (Rasband, W.S., ImageJ, US National Institutes of Health, Bethesda, Maryland).

**Flow cytometry.** Cells were collected with accutase (Sigma) and immediately washed in 1 x PBS. They were then incubated with Triton X-100 (0.1%), RNAse (100 µg/ml) and 7-AAD (559925 from BD Pharmingen) for 1 h at 37°C. Cell cycle analysis was carried out with a FACSCalibur (Becton-Dickinson), and data were analyzed with FlowJo (Tree Star Inc.).

# References:

1. Rouzeau, S. et al. (2012) Bloom's syndrome and PICH helicases cooperate with topoisomerase IIalpha in centromere disjunction before anaphase. *PLoS One* 7, e33905.

2. Savino, T.M., Gebrane-Younes, J., De Mey, J., Sibarita, J.B. & Hernandez-Verdun, D. (2001) Nucleolar assembly of the rRNA processing machinery in living cells. *J Cell Biol* 153, 1097-1110.

3. Illuzzi, G. et al. (2014) PARG is dispensable for recovery from transient replicative stress but required to prevent detrimental accumulation of poly(ADP-ribose) upon prolonged replicative stress. *Nucleic Acids Res* 42, 7776-7792.

4. Contreras-Sanz, A. et al. (2012) Simultaneous quantification of 12 different nucleotides and nucleosides released from renal epithelium and in human urine samples using ion-pair reversed-phase HPLC. *Purinergic Signal* 8, 741-751.

5. Huang, D., Zhang, Y. & Chen, X. (2003) Analysis of intracellular nucleoside triphosphate levels in normal and tumor cell lines by high-performance liquid chromatography. *J Chromatogr B Analyt Technol Biomed Life Sci* 784, 101-109.

6. Neelsen, K.J., Chaudhuri, A.R., Follonier, C., Herrador, R. & Lopes, M. (2014) Visualization and interpretation of eukaryotic DNA replication intermediates in vivo by electron microscopy. *Methods Mol Biol* 1094, 177-208.

7. Neelsen, K.J. et al. (2013) Deregulated origin licensing leads to chromosomal breaks by rereplication of a gapped DNA template. *Genes Dev* 27, 2537-2542.

8. Chabosseau, P. et al. (2011) Pyrimidine pool imbalance induced by BLM helicase deficiency contributes to genetic instability in Bloom syndrome. *Nat Commun* 2, 368.

9. Rao, V.A. et al. (2007) Endogenous gamma-H2AX-ATM-Chk2 checkpoint activation in Bloom's syndrome helicase deficient cells is related to DNA replication arrested forks. *Mol Cancer Res* 5, 713-724.
